# Flexible Wearable Heart Rate Monitoring System and Low-Power Design: A Review

**DOI:** 10.3390/s25164913

**Published:** 2025-08-08

**Authors:** Ciyan Zheng, Chengming Yong, Qi Wei, Fei Qiao

**Affiliations:** 1 School of Automation, Guangdong Polytechnic Normal University, Guangzhou 510640, China; yongchengming@stu.gpnu.edu.cn; 2 Department of Precision Instruments, Tsinghua University, Beijing 100190, China; weiqi@tsinghua.edu.cn; 3 Department of Electronic Engineering, Tsinghua University, Beijing 100190, China

**Keywords:** flexible wearable system, heart rate monitoring, low-power consumption

## Abstract

In an increasingly interconnected world, flexible wearable systems have emerged as transformative technologies, revolutionizing the monitoring and management of personal health and daily activities. With the surging demand for health monitoring, these systems have demonstrated remarkable potential in heart rate monitoring and the detection of heart rate irregularities. This paper provides a comprehensive review of the design of flexible wearable heart rate monitoring systems, with a particular focus on their low-power design. The low-power design is reviewed from four constituent modules of the system, namely the heart rate signal acquisition module, preprocessing module, computation module, and transmission/output module. Meanwhile, for each module, low-power design strategies are reviewed from three different dimensions: hardware-level optimization, algorithm-level enhancement, and hardware–algorithm co-design approaches. Through this multi-dimensional review, the importance of low-power design in flexible wearable heart rate monitoring systems is emphasized. In addition, this paper offers a perspective on the future of low-power design for flexible wearable heart rate monitoring systems. With the advancements in materials science and flexible electronics technology, it is believed that there will surely be better design methods and strategies for the low-power design of flexible wearable systems.

## 1. Introduction

Cardiovascular diseases [1,2] have become the leading causes of death globally. According to the latest WHO reports, they account for about 32% of all deaths in the world. Moreover, heart rate, being a basic physiological sign, is closely related to cardiovascular health. Therefore, it needs to be monitored in real-time and accurately, and abnormal conditions should be detected promptly [3,4,5,6]. Such continuous monitoring can help find diseases early. Consequently, taking timely actions can greatly improve patients’ recovery and the quality of their lives.

The systems mentioned in this paper all refer to complete systems with signal input, signal preprocessing, signal computation, and signal output. To break through the scope of clinical medical applications and integrate heart rate monitoring with various application scenarios such as daily home care, researchers have proposed wearable heart rate monitoring systems [7,8]. A wearable heart rate monitoring system is a portable system integrating technologies such as biological sensing [9], signal processing, and data transmission. Its core function is to acquire heart rate physiological signals in real time through attachment or wearing on the human surface and perform analysis and processing. The emergence of this system has enhanced the flexibility and portability of heart rate monitoring and expanded its application scenarios. Wearable heart rate monitoring can be divided into rigid-body systems and flexible systems. A rigid-body wearable heart rate monitoring system [10,11] refers to a heart rate monitoring system in which the shape of components and the relative positions between internal components remain basically unchanged when the system is in motion or under force. Rigid-body wearable heart rate monitoring systems are widely used in clinical medicine, special industrial environments, and other scenarios due to their strong structural stability and anti-interference capabilities. In contrast, flexible wearable heart rate monitoring systems are widely used in home care, sports health, and other scenarios due to their wearing comfort [12] and lightweight characteristics.

Given the huge potential and commercial value of flexible wearable heart rate monitoring systems in home care, sports health, and other scenarios, this paper mainly focuses on the study of flexible wearable heart rate monitoring systems. A flexible wearable heart rate monitoring system refers to a system that uses flexible electronic technology [13,14] as the core, has bendable and stretchable characteristics, can fit the human surface or clothing, and realizes heart rate monitoring.

Within the context of this paper, “flexible systems” [15] is defined as systems constructed based on flexible substrates or deformable materials, featuring mechanical adaptability (capable of bending, stretching, or folding) while stably performing electronic functions. Its core characteristics lie in the synergy of material selection, structural design, and performance. From the perspective of technical implementation paths, this paper covers two typical forms of flexible systems, with explicit quantitative criteria for differentiation.

Fully flexible systems [16] refer to systems where core components such as sensors, transistors, and circuits are entirely fabricated using organic semiconductors such as polythiophene derivatives, flexible inorganic materials such as ultra-thin silicon films, graphene, or elastic conductive materials [17] such as silver nanowire/PDMS composites. The overall structural thickness is ≤30 µm, enabling ultrahigh mechanical flexibility—maintaining stable electrical performance under conditions of a bending radius ≤ 5 mm and tensile strain ≥ 20% [18]. For example, flexible sensors based on organic semiconductors can achieve structures as thin as 2 µm through molecular-scale thin-film fabrication technology, meeting the conformal characteristics required for skin attachment.

Hybrid flexible systems [19] refer to systems that combine traditional rigid chip components such as silicon-based MCUs, ceramic capacitors with flexible substrates such as polyimide, polydimethylsiloxane through rigid-flexible integration technology. The flexibility of such systems stems from the mechanical adaptability of the substrate, with quantitative standards defined as a flexible substrate thickness of ≤50 µm, an overall achievable bending radius of ≤10 mm, and rigid components accounting for ≤30% of the total area by area ratio [20]. These criteria ensure the system can conform to skin or curved carriers and avoid reduced comfort or conformity failure caused by excessively high rigid component ratios. Examples include the filter module in the flexible system mentioned in this paper, the processor module involved in computing, as well as the BLE module, Wi-Fi module, and Zigbee module and so on required for wireless communication. All these modules can be implemented through rigid-flexible integration technology.

In addition to defining the concept of flexible systems, this paper also elaborates on the optimization of hardware, algorithms, and hardware–algorithm co-design for flexible wearable heart rate monitoring systems. In the hardware design of the system, flexible materials such as polymer materials [21], carbon-based materials [22], and liquid metals [23] are used to achieve the flexibility of the system. These flexible materials have good electrical conductivity and flexibility [24]. By designing their structures [25] (such as wavy or serpentine configurations), the flexible materials can absorb stress through rotation and buckling during stretching, avoiding damage to the conductive paths and improving conformability at the same time [26]. The devices made of these flexible materials have the advantages of high flexibility and comfort, which well solve the shortcomings of traditional systems. In addition to the use of flexible materials in hardware, flexible wearable heart rate monitoring systems also require corresponding algorithm optimization and adaptation. Algorithmic optimization can reduce the hardware load [27] and ensure the stable operation of the system. A typical example is the adoption of a distributed collaborative optimization algorithm [28] in the process of heart rate acquisition, which enables multiple flexible sensing nodes to automatically adjust the sampling frequency and precision during data acquisition, ensuring the integrity of physiological signals while reducing the synchronization error [29] caused by the flexible deformation of hardware. In terms of hardware–algorithm co-design optimization, hardware structure design can accelerate algorithms, while algorithms can reduce hardware burden. As described in Section 2.3 of this paper, when designing application-specific integrated circuits for flexible wearable systems, by designing system-specific circuit structures and combining them with model compression algorithms, the complexity of the flexible system is significantly reduced, the task processing capability of the flexible system is accelerated, and the system power consumption is lowered.

The emergence of flexible wearable heart rate monitoring systems has enhanced the flexibility and comfort of heart rate monitoring systems. More importantly, it has broken through the limitations of usage scenarios [30,31]. Such systems can be widely applied in various scenarios, including daily health management, sports training monitoring [32], and home care for chronic diseases [33]. For example, during marathon events, runners can use flexible wearable systems worn on their wrists to obtain real-time heart rate variability data, thereby optimizing their exercise rhythm. At the same time, the elderly can use washable flexible patches to achieve a 24 h arrhythmia early warning. This continuous monitoring [34] significantly improves the convenience and timeliness of health management, demonstrating the revolutionary value of flexible wearable technology in the field of modern healthcare.

However, the widespread application of flexible wearable heart rate monitoring system is limited by a key issue: the low power issue of the system [35,36,37]. Frequent charging of flexible wearable heart rate monitoring systems not only degrades the user experience but also affects the ability of the system to provide continuous monitoring. This limitation is particularly evident in scenarios requiring uninterrupted data collection, such as medical emergencies or high-intensity sports performance tracking. Additionally, unexpected power outages during critical monitoring periods may lead to the loss of vital physiological data, thereby influencing clinical decision-making processes and posing risks to patient safety. For flexible wearable medical systems, replacing or charging the battery may be difficult or even impossible. Therefore, low-power design [38,39] is not just an extra feature but a necessary condition to ensure reliable system operation, accurate data, and continuous user adoption.

Compared with existing studies on flexible wearable heart rate monitoring systems [12,30], as shown in Table 1, the reasons for selecting these literatures are as follows: first, all of them involve flexible wearable heart rate monitoring systems; meanwhile, they also include descriptions of low power consumption, so they can be compared with the thematic content of this paper.This paper focuses on the low-power design of such systems. The research work is centered around four core components: heart rate signal acquisition, signal preprocessing, signal computation, and signal transmission and output. In the design process of each component, we conduct low-power optimization from three dimensions: hardware-level optimizations, algorithmic energy efficiency improvements, and holistic hardware–algorithm co-design. The research goal is to explore a complete and systematic low-power implementation scheme, providing support for the further development of flexible wearable heart rate monitoring technology.

## 2. Overview of Flexible Wearable Heart Rate Monitoring Systems

Flexible wearable heart rate monitoring systems represent the crystallization of materials science and biomedicine. These systems utilize special flexible materials to fabricate lightweight sensor electrodes and circuits that can conform to the skin. Such a design ensures the systems can acquire stable signals even when the human body is in motion. Meanwhile, biomedical technologies endow these systems with the capability to accurately monitor heart rate and maintain compatibility with the human body. By leveraging biosensing principles [40] to detect subtle physiological signals and employing biomedical algorithms to process the data for calculating clear heart rate values, they enable continuous and precise monitoring services, which are widely applicable to scenarios such as fitness, disease prevention, and health management.

Given the significance of flexible wearable heart rate monitoring systems, this section provides a detailed introduction to such systems, covering their modular composition, modular functions, and the underlying principles of heart rate monitoring. Finally, this section places particular emphasis on the low-power design of flexible wearable heart rate monitoring systems.

### 2.1. Definition of Flexible Wearable Heart Rate Monitoring Systems

**Figure 1:**
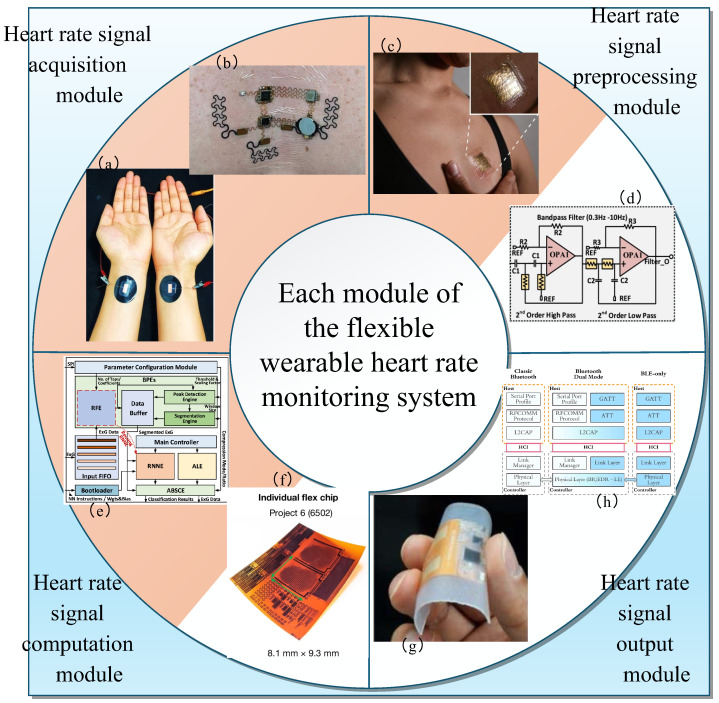
(**a**) Flexible electrodes with sensors [41], under CC BY 4.0, copyright 2020, Springer Nature. (**b**) Flexible ECG sensor [42], under CC BY 4.0, copyright 2023, Wiley Online Library. (**c**) Flexible organic differential amplifiers, reproduced with permission [43], copyright 2019, Springer Nature. (**d**) Band-pass filter circuits, reproduced with permission [44], copyright 2023, Elsevier. (**e**) The BioAIP processor, reproduced with permission [45], copyright 2023, IEEE. (**f**) A flexible chip, reproduced with permission [46], copyright 2024, Nature. (**g**) A BLE module [47], under CC BY 4.0, copyright 2019, IEICE TRANSACTIONS on Information and Systems. (**h**) A BLE protocol stack, reproduced with permission [48], copyright 2023, Elsevier.

In this paper, the flexible wearable heart rate monitoring system is composed of four modules: the heart rate signal acquisition module, the heart rate signal preprocessing module, the heart rate signal computation module, and the heart rate signal transmission and output module, as shown in Figure 1, each with its specific function. Meanwhile, this subsection also introduces the technical principles of the flexible wearable heart rate monitoring system.

The heart rate signal acquisition module primarily employs flexible sensors or signal acquisition circuits to capture cardiac signals for the system [41], such as ECG and PPG signals. During the acquisition process, intelligent algorithms dynamically optimize the sampling rate of these flexible components to ensure optimal data capture. However, the raw signals often contain noise, artifacts, and baseline drift [49]. Consequently, the preprocessing module [43] utilizes filtering circuits and adaptive algorithms to denoise, remove artifacts, and amplify the signals [50], enhancing signal strength. The preprocessed signals are then transmitted to the computation module, which leverages microprocessors [46] and neural network algorithms to perform tasks including heart rate feature detection, heart rate variability (HRV) analysis, and arrhythmia detection [51]. Finally, the processed data is transmitted via low-power Bluetooth (BLE) [48], Wi-Fi, or other wireless protocols to mobile devices or cloud platforms for further analysis and visualization.

The modularity of flexible wearable heart rate monitoring systems offers multiple advantages: First, in terms of hardware, it enables layered integration of flexible materials and functional components [52], allowing tight adherence to the human body’s contours to significantly enhance wearing comfort. Independent power consumption control also extends system battery life. Second, the signal processing flow is more professional—layered filtering and dynamic calibration mechanisms [53] can boost the Signal-to-Noise Ratio to over 35 dB, effectively addressing interference issues like motion artifacts. Third, system maintenance and functional expansion are more flexible [54]: it supports independent replacement and repair of modules, and new functions can be quickly implemented through expansion modules, greatly reducing development and maintenance costs.

**Figure 2:**
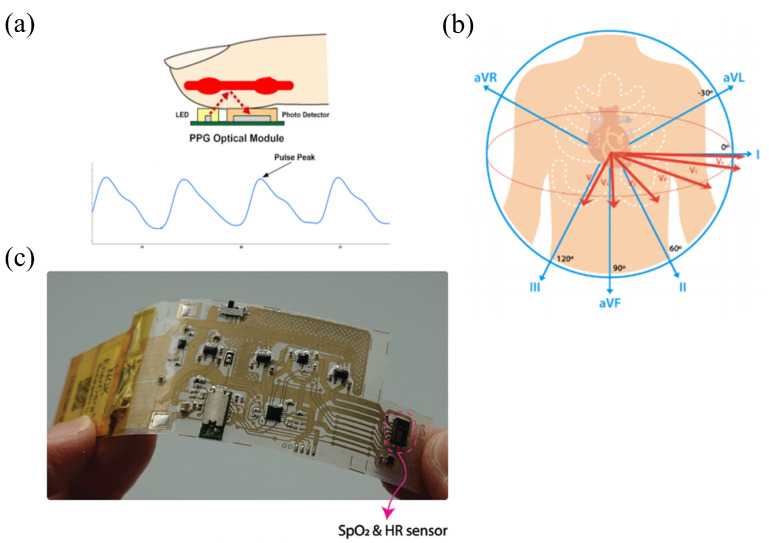
(**a**) Principle and waveform diagram of PPG heart rate monitoring. (**b**) Principle of 12-lead ECG heart rate monitoring. (**c**) The circuit of a flexible hybrid electronic system for heart rate monitoring fabricated by multi-layer screen printing [55]; this image is licensed under CC BY 4.0, copyright 2024, Wiley Online Library.

From a technical perspective, the operation of flexible wearable heart rate monitoring systems mainly relies on PPG and ECG technologies. As shown in Figure 2a,b, PPG sends out light with specific wavelengths onto the skin tissue. By using the way that blood absorbs and reflects light, it changes the changes in blood volume caused by heartbeats into electrical signals. This makes it possible to calculate the heart rate accurately. On the contrary, ECG technology measures the differences in electrical potential on the body’s surface. These differences are caused by the heart’s electrical activity, and through this, an electrocardiogram is obtained. This enables people to analyze the heart rate and the electrophysiological condition of the heart. Compared with PPG, ECG can provide more detailed information about the heart’s electrical activity. Also, it is more sensitive when it comes to detecting heart diseases such as arrhythmias.

In practical applications, these two technologies can play roles in different scenarios. PPG technology enables long-term low-power heart rate monitoring to meet the needs of daily continuous monitoring, while ECG technology plays a key role when a more accurate diagnosis of heart problems is required. However, whether the research is based on ECG technology or PPG technology, its core essentially revolves around the theme of non-invasive monitoring [56]. It can be foreseen that in the future, researchers will be able to develop flexible wearable heart rate monitoring systems with better performance and greater portability.

In summary, the flexible wearable heart rate monitoring system realizes the complete process from heart rate signal acquisition, preprocessing, and computational analysis to signal output through modular design. The modular design significantly enhances the wearing comfort of the system and makes system maintenance and functional expansion more flexible. Meanwhile, the technical principles adopted by the system, namely PPG and ECG, provide diverse and reliable technical support for heart rate monitoring and cardiac health assessment.

### 2.2. Technical Demonstration of Flexible Wearable Heart Rate Monitoring Systems

This subsection demonstrates complete flexible wearable heart rate monitoring systems based on ECG and PPG technologies through several research cases. Thanks to their unique body-conforming designs, these systems can stably monitor heart rates over long periods, breaking through the temporal and spatial limitations of traditional monitoring methods. They exhibit unparalleled advantages, whether in real-time heart rate tracking during daily activities or precise monitoring in special situations.

Voropai et al. [57] developed a flexible wearable heart rate monitoring system based on ECG technology using dry conductive flexible textiles coated with nickel/copper (Ni/Cu). They employed headphones and the dry conductive flexible textiles as carriers to collect ECG signals measured from the mastoid area behind the ears as input signals. These ECG signals were preprocessed through a specially designed instrumentation amplifier circuit and an analog filter, then computationally processed by a 32-bit ARM microcontroller, and finally outputted and stored in an SD card.

Meanwhile, Yoshida et al. [55] developed a flexible wearable heart rate monitoring system based on PPG technology by integrating flexible silver ink circuits through multi-layer screen printing, as illustrated in Figure 2c. The system employs the MAX30102 sensor chip (It is manufactured by Analog Devices, Inc., headquartered in Norwood, MA, USA) to acquire PPG signals as the input and is equipped with an external accelerometer (KX122,which is produced by Kionix, Inc., based in Ithaca, NY, USA) and an external oscillator. By combining specific algorithms, it preprocesses the input signals and compensates for motion artifacts. Subsequently, the sensor hub integrated circuit (MAX32664, it is manufactured by Analog Devices, Inc., Norwood, MA, USA) receives the signals, computes the heart rate value via embedded algorithms, and finally outputs the processed PPG signals to a smartphone through a Bluetooth Low Energy (BLE) module.

In summary, the flexible wearable heart rate monitoring systems described in this subsection, which are based on ECG and PPG technologies, are all composed of four modules: the input of raw signals, preprocessing and computational operations of these signals through internal system modules, and the final output of the processed signals.

### 2.3. Low-Power Design Methods of Flexible Wearable Heart Rate Monitoring Systems

To extend the battery life of flexible wearable systems, a low-power design is crucial. In this paper, the low-power design of flexible wearable systems primarily targets the heart rate signal acquisition module, heart rate signal preprocessing module, heart rate signal computational module, and heart rate signal output module. The low-power design of each module can be further divided into hardware design, algorithm design, and hardware–algorithm co-design.

From a hardware perspective, the hardware power consumption of each module can be significantly reduced by selecting low-power materials and optimizing circuit design. In terms of materials, organic field-effect transistors (OFETs) can be selected. Based on organic semiconductor materials, OFETs can be processed in a solution, and their power consumption is more than 10 times lower than that of traditional silicon devices. For example, Yui et al. [58] reported a glucose biosensor based on OFETs, which has the characteristics of low-power consumption and high sensitivity for glucose detection. Carbon nanotube (CNT) materials have high electron mobility and can replace some CMOS devices, reducing dynamic power consumption. Low-power glucose sensing based on CNT materials [59] has been developed and applied in a series of potential applications. A series of piezoelectric materials, such as PVDF and piezoelectric ceramics [60], can also be selected to achieve self-power supply of the system, thus reducing the power consumption of the system. For instance, Lu et al. [61] reported an electronic tattoo sensor based on PVDF, realizing a self-powered and high-precision sensing module. In terms of circuit optimization, power consumption can be further reduced by simplifying or adjusting the circuit structure and selecting appropriate component parameters. For example, Wu et al. [62] optimized the configuration and physical layout of a resistor–capacitor (RC) network and carefully selected resistor and capacitor values to design an active low-pass filter for PPG signals. This design effectively suppresses noise in PPG signals while minimizing power consumption. Additionally, application-specific integrated circuits (ASICs) [63] with customized architectures can significantly reduce system power consumption.

In hardware-level optimization for low-power design of flexible wearable heart rate monitoring systems, beyond innovations in systems and circuits, non-ideal factors such as contact resistance and wiring resistance play crucial roles that cannot be ignored. These factors, which are less prominent in rigid electronics, become critical in flexible systems due to their mechanical adaptability requirements, directly affecting energy efficiency and signal integrity.

Contact resistance [64] primarily arises at two key interfaces: between flexible electrodes and the skin, and between heterogeneous materials (e.g., rigid chips bonded to flexible substrates). Excessively high contact resistance causes significant signal attenuation, forcing front-end amplifiers to operate at higher gain or bandwidth to maintain the required Signal-to-Noise Ratio. This can increase power consumption by 20–30% [45]. Optimizing electrode materials (e.g., using conductive hydrogels) and the contact area reduces skin–electrode resistance to below 10 kΩ, while adopting low-resistance interconnects lowers interlayer bonding resistance to <5 Ω, minimizing unnecessary power loss in signal transmission [65].

Wiring resistance [66] on flexible substrates (e.g., polyimide or PDMS) is another critical factor, influenced by material conductivity, line dimensions, and mechanical deformation. A 100 µm-wide silver nanowire trace, for instance, may exhibit 5–10 Ω/cm resistance, increasing by 15–20% under 20% tensile strain [67]. High wiring resistance exacerbates IR drops and signal loss, especially for high-frequency heart rate signals, requiring additional power to drive transmission. Mitigation strategies include using high-conductivity composites (e.g., silver nanowire/graphene hybrids), optimizing serpentine wiring geometries to reduce deformation-induced resistance changes, and increasing the thickness of the wire to reduce resistance.

At the algorithm level, low-power design can be implemented according to the functions of each module to ensure low-power consumption while achieving module functionality. In the heart rate signal acquisition module, instead of using a fixed high sampling rate, a dynamic sampling frequency [68] switching algorithm can be employed. This algorithm allows the module to avoid continuous operation in high-frequency regions, thereby reducing overall power consumption. In the signal preprocessing module, by optimizing algorithm parameters [69], such as filter length, step size, and the number of iterations, the required number of repetitive calculations can be reduced under the same denoising requirements, significantly lowering computational load and energy consumption. For the signal computational processing module, model compression is a viable approach. Leveraging lightweight neural network algorithms [70], such as MobileNet or TinyML architectures, can minimize computational power consumption during the inference phase. In the signal output module, employing compression algorithms [71] can reduce transmission power consumption. Simultaneously, optimizing the transmission protocol is another effective way to decrease power usage.

Hardware–algorithm co-design represents a form of deep coupling between hardware architecture and algorithmic characteristics [72], where algorithm optimization reduces hardware load [73] and hardware features enhance algorithm efficiency. Through hardware–algorithm co-design, the power consumption of each module can be effectively reduced. Designing working and sleep modes [74] within modules is a typical example of hardware–algorithm co-design. In the implementation process, sensors can be used to detect changes in the body’s state to enable switching between working and sleep modes. These state signals are then transmitted to a microprocessor, which analyzes the data and employs corresponding algorithms to control the system’s working or sleep states. This approach of achieving mode switching through hardware–algorithm collaboration can effectively reduce overall system power consumption. On the other hand, adopting customized processors and optimized model algorithms [75] is another effective hardware–algorithm co-design method for power reduction. By designing dedicated circuit architectures and applying model compression techniques, system complexity is significantly reduced, while model size and computational load are minimized. The dual optimization of hardware structure and algorithmic efficiency creates a synergistic effect, ultimately lowering the power consumption of wearable systems.

In summary, the low-power design of each module in flexible wearable heart rate monitoring systems requires integrated optimization from three dimensions: hardware, the algorithm, and hardware–algorithm co-design. Hardware optimization reduces module power consumption by selecting low-power materials and piezoelectric materials, as well as optimizing circuit designs such as RC networks and application-specific integrated circuits. Meanwhile, it is necessary to specifically address the contact resistance and wiring resistance issues unique to flexible scenarios—contact resistance is controlled through electrode material optimization and heterogeneous interconnection improvement, while transmission loss is reduced by means of high-conductivity composite materials and structured wiring design. Algorithmic optimization significantly reduces computational load and energy consumption through methods such as dynamic sampling, parameter-tuned filtering, lightweight networks, and transmission compression. Hardware–algorithm co-design ensures the system’s monitoring accuracy while reducing power consumption through approaches like adaptive mode switching and customized processor–model integration. These strategies provide a practical design framework for balancing functionality and energy efficiency in flexible wearable heart rate monitoring systems. The following sections will focus on the theme of low-power consumption to design each module of the flexible wearable heart rate monitoring system.

## 3. Design of the Flexible Wearable Heart Rate Signal Acquisition Module

The heart rate signal acquisition module serves as a critical component in flexible wearable heart rate monitoring systems, providing essential means for obtaining human physiological status. This module can collect ECG and PPG signals through various flexible sensors and perform simple processing on the collected signals. For example, it calculates the optimal sampling frequency via algorithms. Through hardware–algorithm co-design, it realizes efficient signal acquisition compression and sampling compression. The implementation of low-power operation is accomplished through careful material selection, circuit design, and algorithm optimization.

This section will systematically present the design of low-power heart signal acquisition modules from three perspectives: hardware design, algorithm design, and hardware–algorithm co-design approaches.

### 3.1. Low-Power Hardware Design of Flexible Wearable Heart Rate Signal Acquisition Module

In this subsection, we mainly focus on the hardware low-power design of the heart rate acquisition module. In the acquisition of traditional heart rate signals, different kinds of sensors are often used as hardware. These sensors include bioelectric sensors [76], photoelectric sensors [77], and electromechanical sensors [78]. These rigidly designed sensors pose inconveniences during human body signal collection. Additionally, traditional sensors lack consideration for low-power consumption, resulting in high overall energy usage. As flexible electronics technology has progressed and there is an increasing need for flexible sensors in the field of biomedicine, researchers have made improvements and innovations based on traditional sensors, giving rise to a series of low-power flexible sensors [79], such as those made of piezoelectric materials.

For low-power flexible sensors, the rational selection of materials is crucial to achieving low-power consumption. At the same time, measures can also be taken in the hardware layout of sensors, such as adopting multimodal sensing and optimizing hardware design.

**Figure 3:**
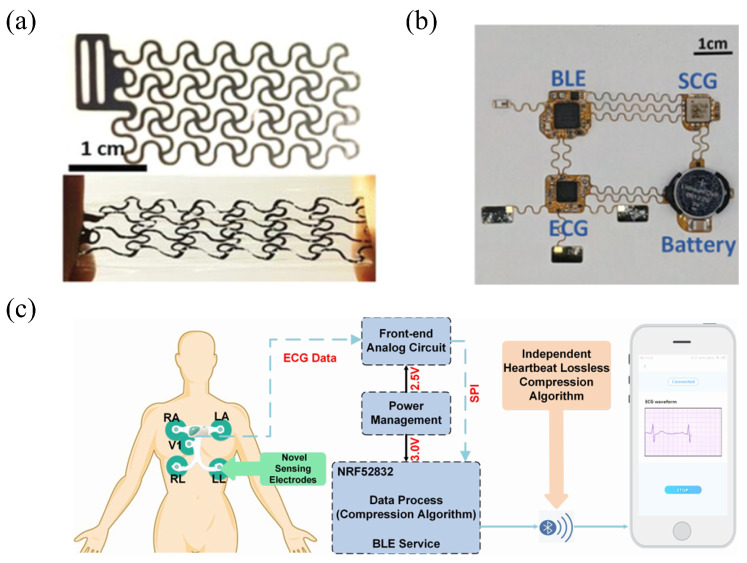
(**a**) Ultra-thin stretchable ECG sensor based on 28-micrometer-thick PVDF material [61]; this image is licensed under CC BY 4.0, copyright 2019, Wiley Online Library. (**b**) Schematic diagram of the structure of an ECG sensor based on graphene materials and electronic tattoo technology [42]; this image is licensed under CC BY 4.0, copyright 2023, Wiley Online Library. (**c**) Schematic architecture framework of the multi-lead ECG acquisition, computation, and transmission system. Reproduced with permission [80], copyright 2022, IEEE.

As reported by Lu et al. [61], as shown in Figure 3a, a heart rate signal acquisition module in a flexible wearable system has been designed; its core component is a low-power sensor based on polyvinylidene fluoride (PVDF) piezoelectric materials. The sensor utilizes 28-micrometer-thick PVDF as the core flexible material. By leveraging the piezoelectric properties of PVDF, the sensor achieves self-powered sensing, directly converting mechanical vibrations into charge signals without the need for an external power supply, thereby reducing energy consumption at the source. Its dual-sensor design suppresses common-mode noise through differential processing of adjacent signals, minimizing power consumption during the post-processing stage. Compared with traditional continuously powered sensors, this self-powered mode and optimized signal processing strategy significantly reduce the overall power consumption.

Furthermore, Lu et al. [42] designed a low-power dual-mode signal acquisition sensor module in the flexible wearable heart rate monitoring system by optimizing the hardware layout of the sensors, as shown in Figure 3b. The system adopts a hardware synchronization mechanism and a time-sharing operation strategy, where each ECG sampling triggers SCG sampling through hardware pulse synchronization, thereby avoiding the computational power consumption caused by subsequent algorithm synchronization. In addition, the system uses a high-stability crystal oscillator (32.768 kHz) to unify the clock, reducing the calibration energy consumption caused by independent clock drift. The total power consumption of the system is approximately 3 mW, and compared with traditional ECG sensors, the power consumption is reduced by more than 40%.

Meanwhile, Lee et al. [81] also developed a low-power, real-time signal acquisition module in the flexible wearable health monitoring system. This system features a hierarchical power supply circuit design that separately powers low-power and high-power modules. The on/off states of high-power modules are controlled by a boost converter and a CMOS switch array, enabling them to activate only when necessary, thereby minimizing system power consumption. To further reduce power usage, the system incorporates hardware such as Nordic’s low-power microcontroller and Texas Instruments’ low-power analog front-end, resulting in a final average power consumption of 16.5 mW for the system.

It is important to emphasize that in the process of achieving low-power consumption, attention must be paid to the relationship between material-based self-powered designs and circuit-level optimization designs, and a rational selection must be made based on application scenarios.

In terms of long-term reliability, although the self-powered characteristics of piezoelectric materials (PVDF) can reduce power consumption at the source—as demonstrated by the 28-micrometer-thick flexible substrate-based PVDF sensor designed by Lu et al., which achieves self-powered functionality [61]—prolonged mechanical cycling may lead to the degradation of the piezoelectric coefficient, thereby affecting the stability of signal acquisition. In contrast, circuit-level optimizations, such as application-specific integrated circuit (ASIC) integration and resistor-capacitor (RC) network tuning [63], are not affected by material fatigue; however, the increased hardware complexity may introduce additional leakage currents, thus requiring a trade-off between stability and power consumption.

In terms of motion scenario and environmental dependence, the output signal strength of self-powered sensors directly depends on the intensity of human motion and is also affected by environmental factors: sweat during strenuous exercise may corrode the electrodes, and high temperatures will accelerate their oxidation. In static scenarios, in addition to possible signal loss due to insufficient energy harvesting, low temperatures will also weaken the conductivity of flexible materials. For example, the dual-mode system developed by Lu et al. [42] needs to activate the auxiliary energy storage module in low-motion states and also start the temperature compensation mechanism in extremely cold environments. Circuit-level low-power strategies such as time-sharing power supply and clock synchronization [82] are less susceptible to environmental interference, but high-frequency vibrations caused by motion may affect timing stability. The parameter drift of passive components caused by extreme temperature and humidity, combined with mechanical stress generated by motion, will further amplify deviations, requiring additional calibration circuits for compensation, which indirectly increases power consumption.

In terms of the complexity of integration with other circuit components, flexible self-powered materials need to achieve molecular-level compatibility with various components in the circuit; otherwise, interface delamination is prone to occur, increasing the difficulty of circuit integration processes [61]. Although the modular design of circuit-level optimizations, such as the hierarchical power supply circuit proposed by Lee et al. [81], reduces the threshold for integration with other circuit components, it may still introduce additional contact resistance, thereby partially offsetting the power consumption optimization effect.

In summary, the realization of low-power flexible sensors is mainly reflected in the rational selection of materials and the hardware layout of sensors. In terms of sensor material selection, these new sensors can adopt self-powered materials, such as PVDF and piezoelectric ceramics, endowing them with ultra-thin characteristics and facilitating low-power design. In terms of sensor hardware layout, strategies such as multi-sensor collaboration and unique peripheral synchronization are adopted to minimize power consumption in the noise processing stage. Meanwhile, the system power consumption can be significantly reduced by selecting low-power components and optimizing the sensor working circuit and power management circuit. Future designs need to further synergize material innovation with circuit optimization. For example, developing ASICs with low leakage based on flexible self-powered materials to break through the existing trade-off bottlenecks.

### 3.2. Low-Power Algorithm Design of Flexible Wearable Heart Rate Signal Acquisition Module

The power consumption of algorithms in heart rate signal acquisition modules is also one of the key factors restricting the low-power development of flexible wearable heart rate monitoring systems. To achieve lower power consumption, there is significant room for improvement in algorithm design. Among these, the adjustment of the sampling rate in signal acquisition modules is crucial to balance power consumption and signal acquisition accuracy.

Bent et al. [68] proposed an algorithm for optimizing the optimal sampling rate of flexible wearable optical sensors through statistical analysis methods. The researchers evaluated the statistically significant differences in PPG and ECG indices at different sampling rates using paired two-tailed *t*-tests (Bonferroni correction), calculated the average bias and 95% limits of agreement using Bland–Altman analysis to measure consistency, and visualized the correlation between ECG indices and PPG errors through linear regression. The optimal sampling rate of wearable optical sensors was determined by synthesizing these three methods. This algorithm significantly reduces data storage volume and costs while lowering system power consumption.

In addition to achieving low-power consumption for the heart rate acquisition module by optimizing the sensor sampling frequency, John et al. also proposed a dynamic heart rate acquisition interval adjustment algorithm [82] based on multimodal data fusion to reduce the power consumption of the flexible wearable heart rate acquisition module. This algorithm for dynamically adjusting heart rate acquisition intervals first uses multimodal data fusion to identify activity states, for example, distinguishing between scenarios such as sedentary and exercise using accelerometers and gyroscopes, before dynamically mapping acquisition intervals based on a preset rule engine or machine learning models. Specifically, the interval is extended to 5 min during sedentary states to save power, while it is shortened to 10 s during exercise to capture rapid heart rate changes. Furthermore, the algorithm can be integrated with dynamic voltage regulation and data compression technologies to reduce energy consumption, ultimately balancing power consumption and real-time performance while ensuring data accuracy.

In summary, in the low-power design of algorithms for flexible wearable heart rate signal acquisition modules, using sampling rate optimization algorithms and dynamic heart rate acquisition interval adjustment algorithms are effective approaches to achieve module low-power consumption. Specifically, these methods enable sensors in the heart rate acquisition module to dynamically adjust the optimal sampling rate and acquisition time based on the user’s activity state. This dynamic adjustment avoids the high-power consumption caused by continuous high sampling rates. As a result, these methods can strike a balance among module power consumption, signal accuracy, and system performance.

### 3.3. Hardware–Algorithm Co-Design of Flexible Wearable Heart Rate Signal Acquisition Module

Hardware–algorithm co-design is one of the important methods to achieve low-power design of flexible wearable heart rate signal acquisition modules. This method can be used to realize efficient heart rate signal acquisition and compressed sampling functions, and it also plays a key role in achieving low-power system design.

Wang et al. [80] reported the signal acquisition module in a low-power flexible wearable heart rate monitoring system is realized through hardware-algorithm co-design, as shown in Figure 3c. In this collaborative framework, the system employs an nRF52832 chip as the control and Bluetooth module, configured to execute a VLC-based Huffman compression algorithm [83,84,85] once per second. When the compressed data size exceeds the original, the system directly transmits the uncompressed data to avoid inefficient computations, thereby reducing power consumption. The compression algorithm minimizes computational complexity through reference vector methods and byte grouping, adapting to the MCU’s processing capabilities. Concurrently, the ADS1293 front-end chip (It is manufactured by Texas Instruments Incorporated, with its headquarters located in Dallas, TX, USA) is set to a 400 Hz sampling rate to meet the algorithm’s requirements for high-resolution signals, enabling 3-channel lead acquisition to reduce the hardware channel count. At a 400 Hz sampling rate, the average power consumption of the system is 13.468mW, representing a 16.5% reduction compared to systems without compression algorithms; it outperforms similar single-lead devices.

Similarly, Parvez et al. [63] developed a low-power signal acquisition module in a flexible wearable system through hardware–algorithm co-design. The ASIC in this system measures 4.0 × 2.5 mm^2^. The system starts with an LED driver, which is connected to a photodiode (PD) through a current path. The current generated by the photodiode is converted into a voltage signal by a transimpedance amplifier (TIA). The voltage signal then passes through a low-pass filter (LPF) to remove high-frequency noise. The filtered signal enters an analog-to-digital converter (ADC) to convert the analog signal into a digital signal. Finally, the digital signal is processed by a digital baseband engine (DBE), and the PPG reading is output. Specifically, the analog front-end (AFE) of the system dynamically adjusts the gain and bandwidth through 16 digital input/output pins to meet the sparse sampling requirements of the compressive sensing algorithm and ensure the retention of signal features. The compressive sensing algorithm reduces the sampling rate to 4 Hz, significantly reducing the amount of hardware data processing and avoiding the need for high-frequency operation of the ADC. In addition, the LED drive circuit controls the current through a 4-bit analog switch and adjusts the compression ratio of the compressive sensing algorithm to dynamically optimize the light intensity for power reduction. The heart rate extraction algorithm omits the signal reconstruction step and directly outputs 8-bit heart rate data, thereby reducing the requirements for transmission bandwidth. Finally, the algorithm controls the microcontroller to enter low-power mode according to the operating state of compressive sensing and cooperates with Bluetooth Low Energy (BLE) to transmit data every 40 s, optimizing the total system power consumption to 1.66 mW.

In summary, as demonstrated by the case studies of Wang et al. and Parvez et al., hardware–algorithm co-design is a crucial approach to achieving low-power heart rate signal acquisition. Wang et al.’s research illustrates how dynamic compression algorithms and sampling rate adaptation form a closed-loop optimization, while Parvez’s study proves the effectiveness of sparse sampling and cross-layer parameter tuning in reducing power consumption. These implementations collectively validate that combining low-power hardware components such as ASICs and nRF52832 with intelligent algorithms such as compressive sensing and Huffman coding can systematically address the trade-offs among sampling resolution, data transmission, and energy efficiency. As evidenced by the “high sampling rate-efficient compression–low-power” feedback mechanism, hardware–algorithm co-design has become an indispensable strategy for promoting next-generation wearable medical devices to balance technical feasibility and energy sustainability.

The Table 2 lists the innovativeness, practicality, power consumption optimization methods, power consumption levels, and comparisons of power consumption with previous studies for each technology, as shown in Table 2. Meanwhile, since the two methods mentioned in the algorithm aspect are relatively similar, only one of them is included in the table.

It is worth noting that the literatures [42,63,68,80,81] all focus on research related to flexible wearable systems.

In conclusion, this section systematically demonstrates the multi-dimensional strategies for achieving low-power operation in flexible wearable heart rate signal acquisition modules through hardware design, algorithm design, and hardware–algorithm joint design. In terms of hardware design, self-powered materials such as PVDF and piezoelectric ceramics are selected, the circuit structure is optimized, and multi-sensor collaboration is implemented, and a trade-off analysis is conducted between the low power consumption achieved based on the self-powered nature of materials and that achieved through circuit optimization. In algorithm design, energy consumption is reduced by optimizing the sampling rate and dynamically adjusting the acquisition time. More importantly, in hardware–algorithm co-design, the deep integration of hardware selection and algorithm innovation significantly reduces the module’s power consumption. These comprehensive solutions not only balance the power consumption and performance of the module but also pave the way for the widespread application of energy-efficient flexible wearable heart rate monitoring systems.

## 4. Design of the Flexible Wearable Heart Rate Signal Preprocessing Module

In modern physiological signal monitoring, heart rate signal preprocessing [86] plays a critical role. Following signal acquisition, preprocessing operations are essential to effectively eliminate noise interference, motion artifacts [87], baseline drift, and achieve data normalization. Proper preprocessing not only establishes a reliable foundation for subsequent heart rate calculations but also significantly reduces system processing complexity, thereby contributing to lower overall power consumption.

This section will explore the design methods of low-power heart rate signal preprocessing modules from three key dimensions: hardware design, algorithm design, and hardware–algorithm co-design. Each part will demonstrate how researchers achieve system power consumption optimization while ensuring the integrity of preprocessing functions.

### 4.1. Low-Power Hardware Design of Flexible Wearable Heart Rate Signal Preprocessing Module

Heart rate signal preprocessing primarily encompasses filtering, noise reduction, signal amplification, baseline drift correction and so on. In this section, we focus on the hardware low-power design of the heart rate signal preprocessing module from the perspective of filtering. As the first step in signal preprocessing, filtering directly influences the energy consumption of subsequent processing units, and its importance is undeniable. To achieve hardware low-power design, various miniaturized filtering circuits [88], such as low-pass filters, high-pass filters, and band-pass filters, are typically integrated onto flexible printed circuit boards (FPCBs) to perform critical signal processing functions.

**Figure 4:**
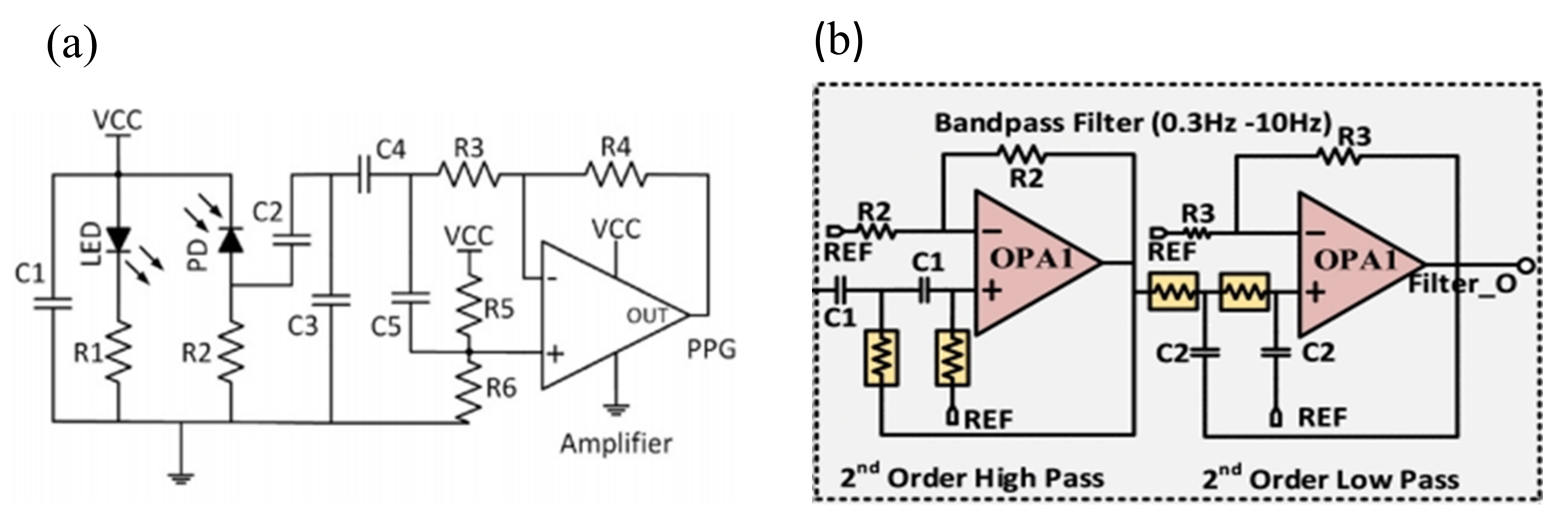
(**a**) Active low-pass filter for PPG signal conditioning. Reproduced with permission [62], copyright 2020, IEEE. (**b**) Circuit with a Band−pass Filter. Reproduced with permission [44], copyright 2021, IEEE.

The application of flexible low-pass filter circuits [89] module is particularly effective in suppressing high-frequency noise, which usually originates from sources such as environmental electromagnetic interference (EMI). These circuits operate on the fundamental principle of allowing low-frequency signals to pass unimpeded while significantly attenuating high-frequency components. As shown in Figure 4a, Wu et al. [62] designed a low-power low-pass filter suitable for PPG signals by optimizing the resistor-capacitor (RC) configuration. Specifically, they optimized the RC network structure and circuit layout of the low-pass filter. By customizing the selection of resistor and capacitor parameters, the cutoff frequency of the filter was precisely set within the optimal range, effectively filtering out high-frequency noise in PPG signals. After calculation, its average power consumption is approximately 10 µW, which is 40% lower than that of traditional filters with fixed resistor and capacitor values, while the noise suppression ratio of PPG signals is increased by 35 dB.

In addition, flexible high-pass filter circuits [90] module can eliminate low-frequency interference in heart rate signals, such as those caused by human respiration, slight limb movements, environmental and temperature changes. The elimination of such interference ensures signal stability. Compared with traditional high-pass filters that use fixed-parameter components, the high-pass filter circuit incorporating adjustable resistors and capacitors can dynamically adjust power consumption according to actual signal conditions while ensuring interference removal, thereby further reducing ineffective energy consumption.

The flexible band-pass filter circuits [91] module combine the advantages of both low-pass and high-pass filters, allowing only signals within a specific frequency range to pass through. They can accurately retain the effective frequency band of heart rate signals while suppressing interference from other frequency bands. As shown in Figure 4b, Pandey et al. [44] realized the design of a low-power band-pass filter through two key aspects: hardware selection and circuit design. Firstly, in terms of hardware selection, they used adjustable pseudo-resistors to replace high-power-consuming components. Compared with the high-power fixed resistors used in traditional band-pass filters, this approach significantly optimized power consumption. Meanwhile, the circuit structure combines a traditional second-order RC low-pass filter with a high-pass filter, whereas the circuit structure of traditional band-pass filter designs is often more complex, leading to higher power consumption. Specifically, by using adjustable pseudo-resistors and specific capacitors, they precisely adjusted the cutoff frequency, which not only removed high-frequency noise in heart rate signals but also alleviated low-frequency interference, thereby reducing additional power consumption. The total system power consumption is approximately 460 µW. With the same noise suppression effect, this design reduces power consumption by about 30% compared with unoptimized band-pass filters.

In practical flexible wearable systems, the effectiveness of hardware filtering strategies is highly contingent on scenario-specific constraints. A comprehensive evaluation integrating the noise characteristics, resource limitations, and performance requirements of the intended usage scenario is essential, rather than simply promoting a single method. The following is an assessment of the applicable scenarios for the three aforementioned flexible filter modules:

Optimized low-pass filter modules excel in suppressing high-frequency noise, such as electromagnetic interference. They offer distinct advantages in heart rate monitoring scenarios under static conditions, where signals are stable and low-frequency drifts are negligible. However, their limitations become prominent in motion scenarios: due to their inability to handle low-frequency interferences like baseline drift, the signal quality in dynamic environments will significantly decline. Consequently, they are unsuitable for long-term dynamic heart rate monitoring or high-intensity exercise scenarios [89].

Tunable high-pass filter modules achieve adaptive suppression of low-frequency baseline drift through dynamic parameter adjustment. This makes them particularly practical for heart rate signal preprocessing in moderate-intensity exercise scenarios. It should be noted, however, that the introduction of tunable components increases circuit integration complexity. Additionally, some tunable components are sensitive to ambient temperature, which may lead to performance degradation in extreme temperature scenarios. Therefore, their applicability is limited in resource-constrained microdevices or scenarios involving operation in special temperature zones [90].

Low-power band-pass filter modules integrate the advantages of both low-pass and high-pass filters, being capable of simultaneously suppressing high-frequency electromagnetic interference and low-frequency baseline drift. They perform optimally during intense exercise in complex environments. However, their power consumption is significantly higher than that of single low-pass or high-pass filters, and their complex circuit structure increases the difficulty of integration onto flexible substrates. This means they are better suited for scenarios with high precision requirements but relatively relaxed power budgets, such as medical-grade exercise monitoring, rather than ultra-low-cost systems or miniature flexible wearable systems [91].

In summary, the aforementioned flexible filtering circuit modules have fully considered low-power characteristics in their design. By selecting low-power electronic components, such as adjustable pseudo-resistors, optimizing the parameter configuration of components like resistors and capacitors, and adopting optimized circuit structures, these circuits significantly reduce energy consumption while ensuring the functionality of the module compared with traditional unoptimized filter designs. This not only extends the battery life of flexible wearable systems but also helps ensure the long-term stable operation of these systems. Meanwhile, when selecting or designing filters, it is necessary to make choices or designs based on the specific usage scenarios or constraints of heart rate monitoring to give full play to the advantages of various filters.

### 4.2. Low-Power Algorithm Design of Flexible Wearable Heart Rate Signal Preprocessing Module

In terms of low-power algorithm design, this paper takes the preprocessing of ECG signals and PPG signals as examples for illustration.

Preprocessing of ECG Signals:

For the preprocessing of ECG signals, an adaptive filtering algorithm is usually employed to remove artifact noise. Take the Least Mean Square (LMS) [92] algorithm, which is currently the most widely used, as a typical example. Sharma et al. [93] proposed a Wiener filtering and adaptive LMS algorithm for ECG denoising in flexible wearable heart rate monitoring systems. Based on the steepest descent method, this algorithm performs excellently in dynamic signal environments. By iteratively adjusting the filter coefficients according to the error between the noisy ECG input and the desired output, using the current error and input signals combined with a step-size factor, it converges to the optimal filtering state, thereby reducing system power consumption. Meanwhile, this algorithm features low computational complexity, easy implementation, and high real-time performance.

Based on the LMS algorithm, researchers have developed the Normalized Least Mean Square (NLMS) algorithm, which has a better filtering effect and lower power consumption. As demonstrated in the research by Saxena et al. [69], they optimized three critical parameters of the Normalized Least Mean Square (NLMS) algorithm—filter length, step size, and iteration count—to develop a power-efficient adaptive filtering method that significantly enhances the performance of ECG denoising in flexible wearable systems. By optimizing these parameters, the system achieves low-power operation without compromising signal integrity. Specifically, optimizing the filter length reduces the number of multiply-accumulate operations required during real-time processing, while adjusting the step size and iteration count minimizes computational load and energy consumption by decreasing redundant iterations under the same denoising requirements.

Compared with the LMS algorithm, the NLMS algorithm has a faster convergence speed. This is because its normalized step size can dynamically adjust the amplitude of the weight update according to the power of the input signal. When dealing with complex signals such as ECG signals, in the face of noise interference and signal fluctuations, it can quickly approach the optimal solution and reduce the time consumption of convergence. Therefore, it can reduce the power consumption during operation. This is attributed to the fact that the NLMS algorithm effectively suppresses large-amplitude noise interference caused by motion through input signal power normalization (Equation (1)) [69]:

(1) $$\mu \left(n\right)=\frac{\mu_{0}}{{∥x\left(n\right)∥}^{2}+\delta }.$$

Equation (1): normalized step-size adjustment of the NLMS algorithm, where $\mu_{0}$ is the initial step size, x(n) is the input signal, and δ is the regularization parameter to prevent division by zero.

Meanwhile, this paper also evaluates the robustness of different algorithms under various motion states. As shown in Table 3, two typical scenarios—resting and running—are compared, with the Mean Square Error (MSE) and Signal-to-Noise Ratio (SNR) used as performance metrics to assess filtering effectiveness. A smaller MSE and larger SNR indicate better performance. According to Table 3, references [94,95] present a comparison between the LMS adaptive filtering algorithm and the NLMS adaptive filtering algorithm, while references [96,97] provide a comparison between wavelet filtering (WT) and Kalman filtering (KF), moreover, references [94,96] focus on research related to wearable systems.WT exhibits superior anti-noise performance in the running state (MSE = 0.065, SNR = 22.3 dB) but with higher power consumption. KF performs similarly to LMS in the resting state (MSE = 0.023, SNR = 27.9 dB) but shows slightly poorer performance in high-dynamic running scenarios (MSE = 0.095, SNR = 19.5 dB) [96,97]. Adaptive filtering (LMS/NLMS) achieves a better balance between performance and low-power consumption. Specifically, the LMS algorithm yields an MSE = 0.021 and an SNR = 28.5 dB in the resting state, and an MSE = 0.089 and an SNR = 20.1 dB in the running state. In contrast, the NLMS algorithm exhibits an MSE = 0.019 and an SNR = 29.2 dB in the resting state, and an MSE = 0.078 and an SNR = 21.0 dB [94,95] in the running state. These results indicate that adaptive filtering algorithms are more suitable for flexible wearable system scenarios.

In addition to the impact of motion states on algorithms, this paper also considers the quantifiable effects of environmental factors on algorithm performance, as shown in Table 4. References [98,99] present a comparison between the LMS attenuation rate and the NLMS attenuation rate, while references [96,100] provide a comparison between the WT attenuation rate and the KF attenuation rate. Among these, references [96,98] focus on research related to wearable systems. When skin humidity increases to 40% RH, the Total Harmonic Distortion (THD) attenuation rates of the LMS algorithm and the NLMS algorithm are extremely high, being 85.7% and 60.7% [98,99], respectively, while the THD attenuation rates of the Wavelet Transform (WT) algorithm and the Kalman Filter (KF) algorithm are relatively low, being 25.0% and 32.1% [96,100], respectively, indicating better performance. When the temperature rises to 35 °C, the Signal-to-Noise Ratio (SNR) attenuation rates of the LMS algorithm and the NLMS algorithm are −18.2% and −12.5% [98,99], respectively, while the SNR attenuation rates of the Wavelet Transform (WT) algorithm and the Kalman Filter (KF) algorithm are −8.7% and −7.3% [96,100], respectively. Among them, the Kalman Filter (KF) algorithm has the lowest SNR attenuation rate and shows the best performance. Similarly, in the case of increased exercise intensity, by comparing the data of each algorithm in the table, it can be known that the Bit Error Rate (BD) attenuation rate of the Wavelet Transform (WT) algorithm is the lowest, indicating the best performance. Overall, different algorithms have different performance under different environmental conditions. The Kalman Filter (KF) algorithm performs prominently when the temperature increases, while the Wavelet Transform (WT) algorithm performs better when humidity increases and exercise intensity increases.

In summary, for ECG signal preprocessing, in addition to adaptive filtering algorithms, such as LMS and NLMS, methods such as WT [101] and KF [102] can also be employed. Each of these algorithms has its own advantages and disadvantages in ECG signal preprocessing. In practical applications, it is necessary to reasonably select or combine these algorithms based on motion scenarios, environmental factors, noise characteristic requirements, and system resource limitations. This approach ensures efficient and accurate preprocessing of ECG signals while taking into account the design of the system’s power consumption.

Preprocessing of PPG Signals:

Currently, many researchers have proposed different methods for acquiring, removing, or lessening the impact of motion disturbances in the PPG signals of flexible wearable systems and have used time-domain and frequency-domain signal preprocessing techniques, as well as machine learning-based methods, to estimate heart rates. These algorithms can mainly be classified into two categories. One is the classical signal preprocessing method, and the other is mainly a deep learning-based method.

The classical signal preprocessing methods mainly include the CurToSS [103] algorithm and the TAPIR [104] algorithm.

As proposed by Zhou et al. in the reference [103], the CurToSS algorithm, designed for the preprocessing of heart rate signals in flexible wearable systems, utilizes the sparse spectral decomposition of photoplethysmogram (PPG) signals to identify spectral onset points and track heart rate trajectories. When signal discontinuities are detected, the algorithm triggers a multi-stage reconstruction process. It distinguishes motion artifacts from heart rate components through constrained spectral searches and cross-spectral analysis. Crucially, to achieve low-power operation, CurToSS optimizes the definition of the search range in curve reconstruction to reduce unnecessary computational load. Meanwhile, an adaptive parameter selection algorithm based on real-time data features replaces the experience-based parameter settings. This approach reduces computational complexity while maintaining performance, thereby enabling low-power operation.

The second method reported by Zhang et al. [104] for flexible wearable heart rate monitoring systems is called the TAPIR algorithm. This algorithm achieves low-power consumption by optimizing three cascaded modules: signal decomposition, sparse signal reconstruction, and spectral peak tracking. Specifically, during the signal decomposition stage, the TAPIR algorithm simplifies the screening process of motion artifacts (MA) in singular spectrum analysis (SSA) [105], reducing unnecessary data processing and thus contributing to power reduction. In the sparse signal reconstruction stage, further optimization of the basis matrix pruning strategy improves computational efficiency, effectively reducing power consumption as well. Additionally, in the spectral peak tracking step, the improvement of the method for judging heart rate continuity reduces complex calculations, creating conditions for low-power operation.

However, when using classical signal preprocessing methods, as the accuracy of the algorithm improves, the classical signal preprocessing methods are usually accompanied by an increase in the number of free parameters, which is not helpful for achieving low-power consumption. In recent years, some researchers have begun to explore deep learning methods for PPG-based heart rate monitoring.

Essalat et al. [99] proposed a supervised learning algorithm based on a low-power neural network (NN) in the reference, which is used for the preprocessing of PPG signals in flexible wearable systems. This algorithm achieves low-power consumption by optimizing stages such as candidate peak selection and feature extraction. The algorithm employs low-power optimization strategies at multiple stages: in the candidate peak selection stage, it can effectively filter out redundant PPG signals collected by flexible wearable systems, thus significantly reducing computational overhead and power consumption; in the feature extraction stage, it accurately identifies key features while eliminating redundant information; and compared with complex architectures, the three-layer multi-layer perceptron (MLP) network minimizes the consumption of computational resources while ensuring high accuracy. By optimizing the computational process to reduce power consumption, this algorithm lays a solid foundation for subsequent research.

In summary, in the preprocessing of heart rate signals acquired by flexible wearable systems, whether it is the adaptive filtering algorithms for ECG signals, such as the LMS algorithm and its optimized version, the NLMS algorithm, the classic algorithms for PPG signal processing, like the CurToSS and TAPIR algorithms, or the emerging deep learning algorithms, they all focus on improving performance metrics such as accuracy while attaching great importance to low-power optimization. By means of improving calculation methods, streamlining parameter adjustment, and optimizing network structures and processes, these algorithms strive to achieve efficient and low-power heart rate signal preprocessing in resource-constrained flexible wearable systems. This not only ensures effective signal processing but also lays a solid foundation for long-term and stable heart rate monitoring as well as subsequent medical applications.

### 4.3. Hardware–Algorithm Co-Design of Flexible Wearable Heart Rate Signal Preprocessing Module

In addition to achieving low-power signal preprocessing operations through the aforementioned hardware and algorithm optimizations, this subsection will introduce more efficient heart rate signal preprocessing via hardware–algorithm co-design.

Shu et al. [106] developed a low-power module for heart rate data recognition in flexible wearable systems through hardware–algorithm co-design. In the heart rate data preprocessing stage, the heart rate sensor and microcontroller unit (MCU) provide stable raw heart rate data and time synchronization support for the algorithm. The algorithm, aiming at the characteristic that PPG sensors are vulnerable to motion artifacts, performs normalization with neutral data as the baseline to reduce the impact of hardware acquisition noise. At the low-power optimization level, the system employs low-power PPG sensors and the Bluetooth Low Energy (BLE) protocol, reducing energy consumption through dynamic adjustment of LED brightness and implementation of a sensor sleep mechanism. On the algorithm side, process optimization is carried out: only valid signal segments are transmitted during data transfer, and lightweight moving average filtering is used to minimize computational load and avoid high-power operations. By combining low-power PPG sensors with lightweight moving average filtering, the average power consumption of the system is approximately 14.4 mW, the preprocessing power consumption is reduced by 25%, and the motion artifact suppression rate is improved by 60%.

Similarly, Fernandes et al. [107] proposed an efficient heart rate data preprocessing module in flexible wearable systems through a hardware–algorithm co-design method. In this system, the hardware GPU undergoes circuit configuration optimization for the Neural-ODE algorithm model to adapt to the computing capability and power consumption constraints of low-power hardware, thereby enhancing the algorithm’s operational efficiency on edge devices. The Neural-ODE algorithm, in turn, simplifies the network structure for hardware adaptation, reducing the hardware load. Meanwhile, it performs min–max normalization on heart rate data, efficiently converting large volumes of data into a unified scale to minimize computational complexity and thus reduce overall system power consumption.

In summary, the hardware–algorithm co-design for low-power consumption in the heart rate signal preprocessing module has achieved advancements. Through the integration of hardware optimization strategies like the adoption of low-power PPG sensors, dynamic adjustment of LED brightness, and the implementation of sensor sleep mechanisms, alongside algorithmic innovations, such as lightweight moving average filtering, data normalization with neutral baselines, and hardware-adapted Neural-ODE model simplification, the system effectively balances signal processing accuracy and power efficiency. These co-design approaches not only address the challenges of motion artifacts and hardware acquisition noise but also minimize computational load and energy consumption by tailoring algorithms to hardware capabilities and vice versa.

The Table 5 lists the innovativeness, practicality, power consumption optimization methods, power consumption levels, and comparisons of power consumption with previous research results for each technology, as shown in Table 5. In the algorithm aspect of the heart rate signal preprocessing module, due to the large number of involved algorithms, this table only selects one traditional algorithm [69] and one neural network algorithm [99] for list. In the algorithm–hardware co-design aspect, since both references [106,107] focus on the normalization operation in the heart rate preprocessing process, this table only selects one of them for list.

It should also be noted that due to the similarities between flexible wearable heart rate monitoring systems and wearable heart rate monitoring systems in terms of method design for heart rate preprocessing, such as both being able to adopt adaptive filtering algorithms, wavelet filtering, etc., and application scenarios [108], some references in this section [93,94,96,97,98] have adopted the preprocessing algorithms of wearable heart rate monitoring systems and migrated them to the relevant research on flexible wearable systems.

In conclusion, this section comprehensively explores the design concepts of low-power heart rate preprocessing modules from three dimensions: hardware design, algorithm design, and hardware––algorithm co-design. Whether it is the adoption of low-power components and optimized circuits at the hardware level such as the design of resistor–capacitor network structures, the use of adjustable pseudo-resistors, etc., the reduction in computational load and energy consumption through technical improvements at the algorithm level such as LMS and NLMS algorithms with optimized parameters and steps and lightweight neural network algorithms, or the innovative combination of hardware and algorithms such as the MCU providing stable raw heart rate data and time synchronization support for the algorithm; the algorithm, targeting the hardware, performs normalization with neutral data as the baseline to reduce the impact of hardware acquisition noise, the core goal of these design approaches is to achieve efficient and low-power heart rate signal preprocessing. It should also be noted that in the algorithm section, the comparison of the robustness of various filtering algorithms under different motion scenarios and environmental influences indicates that when designing system algorithms, it is necessary to consider the application scenarios or environmental constraints of the system and select appropriate algorithms based on the applicable conditions of different algorithms.

## 5. Design of the Flexible Wearable Heart Rate Signals Computation Module

The heart rate signal computation module performs operations such as feature point detection, heart rate abnormality detection, and heart rate variability analysis on the preprocessed heart rate signals.

In this section, we will explore how the heart rate signal computation module achieves low-power design while ensuring its functionality. In hardware design, low-power and high-computational-efficiency microprocessors are selected, and the circuit layout is optimized to minimize unnecessary energy consumption. In terms of algorithm design, complex neural network algorithms are employed to ensure the accuracy of signal processing while reducing computational load and thus power consumption. Additionally, hardware–algorithm co-design should give full play to the advantages of both, coordinating their operation to achieve low-power performance. The following sections will elaborate on these key design points in detail.

### 5.1. Low-Power Hardware Design of the Computation Module for Flexible Wearable Heart Rate Signals

From a hardware perspective, efficient microcontroller units (MCUs) are typically employed for heart rate computation and processing. However, to achieve low-power operation of the MCU, researchers need to meticulously design the MCU and circuit structure according to system requirements.

**Figure 5:**
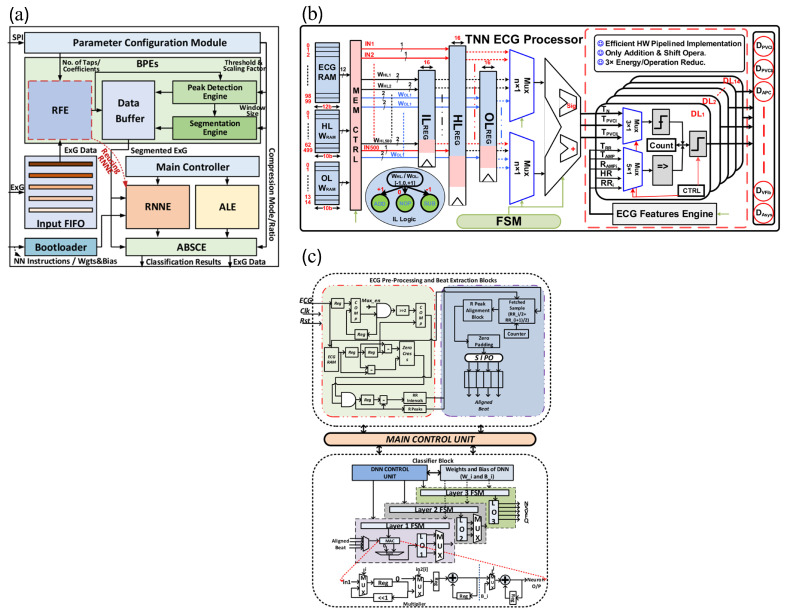
(**a**) Heart rate calculation processor based on the BioAIP structure. Reproduced with permission [45], copyright 2023, IEEE. (**b**) The electrocardiogram processor circuit, based on the ternary neural network. Reproduced with permission [67], copyright 2022, IEEE. (**c**) Schematic framework of the electrocardiogram computing and processing structure based on the deep neural network (DNN). Reproduced with permission [109], copyright 2022, IEEE.

Liu et al. [45] developed the low-power BioAIP processor module for flexible wearable health monitoring through a reconfigurable design. Its system framework is shown in Figure 5a. The BioAIP combines a reconfigurable neural network engine (RNNE) with five parallel processing elements and optimized SRAM units to support multi-mode neural operations. The processor innovatively reuses RNNE resources for FIR filtering by storing coefficients in the existing weight/bias SRAM, reducing hardware overhead. The reconfigurable design curbs hardware overhead and leakage power. The pre-classification circuit reduces the activation frequency of the CNN, and the approximate data compression technology significantly reduces the energy consumption of memory access. Ultimately, the average power consumption of the entire system is approximately 46.8µW, making it the lowest-power system of its kind.

Abubakar et al. [67] developed an ultra-low-power ECG processor module for flexible wearable systems based on ternary neural networks [65] by optimizing the processor design, and its system framework is shown in Figure 5b. In the optimization of the processor, the ternary weight adjustment of the ternary neural network (TNN) reduces storage and power consumption, the pipeline design improves efficiency, and the probability-estimated sigmoid function reduces on-chip consumption. Meanwhile, the TNN-DL hybrid micro-classifier cuts down the amount of computation and storage, and the optimized moving average (MVA) filtering simplifies operations. The processor adopts 65 nm CMOS technology and 2.5 V thick-gate transistors to minimize leakage current and static power consumption, requiring only 1 KB of on-chip memory and consuming as low as 746 nW.

In summary, from the BioAIP proposed by Liu et al. to the ternary neural network-based ECG processor by Abubakar et al., we have witnessed multi-level hardware optimizations through circuit structures. These hardware design methods not only ensure the functionality of the heart rate calculation module but also effectively achieve the goal of low-power consumption through innovative approaches, such as constructing reconfigurable hardware designs, adopting pipeline designs, and implementing event-driven designs.

### 5.2. Low-Power Algorithm Design of the Computation Module for Flexible Wearable Heart Rate Signals

In addition to hardware design, the low-power design of algorithms for the heart rate signal computation module is also of great significance. At the same time, neural network technology has demonstrated great potential in improving the accuracy and efficiency of heart rate computation.

Next, this subsection will explore how to leverage traditional heart rate computation algorithms and neural network algorithms to achieve a low-power algorithm design for the heart rate signal computation module while also balancing the accuracy of heart rate signal computation. Traditional algorithms refer to algorithms that, distinct from neural network algorithms, are based on explicit mathematical models, rely on manually designed features, have low computational complexity, and offer strong interpretability [110].

In terms of traditional heart rate computation algorithms, Rajaby et al. [111] proposed a low-power statistical FFT signal analysis method for flexible wearable systems, which determines heart rate by counting the occurrences of indices within the cardiac frequency range after FFT transformation. In practical applications, clearly defining statistical ranges and presetting appropriate heart rate frequency search intervals according to specific application scenarios can effectively reduce unnecessary statistical computations, resulting in a system power consumption as low as 2.4 milliwatts. Compared with traditional FFT signal analysis algorithms, its power consumption is reduced by more than 40%. Additionally, optimizing the storage method of statistical results by reducing storage space occupancy and minimizing read/write operations also helps to save system power consumption.

Furthermore, Li et al. [112] proposed a low-power Maximum Likelihood (ML) prediction algorithm for flexible wristband systems, which estimates heart rate by constructing and maximizing likelihood functions. During algorithm optimization, approximation methods are employed to simplify complex likelihood function computations and reduce the execution frequency of computationally intensive operations, thereby reducing power consumption while maintaining an acceptable level of precision loss. Moreover, in the iterative optimization process, rational configuration of iteration termination criteria avoids excessive iterations, thereby eliminating redundant computations and ultimately achieving the goal of power reduction.

In addition, in traditional algorithms, the Pan–Tompkins Algorithm and Threshold Detection Algorithm are also very classic heart rate computation methods. For example, Wu et al. [113] proposed an optimized Pan–Tompkins Algorithm. By optimizing the QRS complex localization method, adopting adaptive threshold and backtracking technology, etc., it can quickly locate the P-wave position and accelerate the heart rate calculation speed, thereby reducing the system power consumption to 0.25 mW. Fariha et al. [114] proposed a Threshold Detection Algorithm, which uses a dual-threshold mechanism and dynamically adjusts the thresholds according to signal characteristics to adapt to different heart rate signal scenarios. The high threshold (THR1) is used for initial signal analysis. If no QRS complex is detected within a specific time, the low threshold (THR0) is activated, and the threshold is adaptively updated through historical peak data, resulting in a low system operating power consumption of 0.18 mW.

Traditional heart rate calculation algorithms, such as the Pan-Tompkins Algorithm and Threshold Detection Algorithm, often rely on manually designed feature extraction rules. They have poor adaptability to complex environments, tend to suffer from decreased heart rate monitoring accuracy in complex dynamic scenarios, and lack flexibility. In contrast, neural networks, with their strong self-learning capabilities, can autonomously mine deep-seated features from raw signals and possess excellent adaptive adjustment mechanisms. They can better cope with diverse heart rate signal fluctuations and environmental interference, and exhibit high flexibility, thus currently achieving significant progress in the field of heart rate signal computation.

However, different neural networks are applicable to different scenarios. For example, lightweight Support Vector Machine (SVM) classifiers are only suitable for scenarios with limited heart rate data, minimal noise interference, and single-task requirements, so not all neural networks are applicable to heart rate monitoring in long-term complex scenarios. Some applicable scenarios of neural networks are presented below.

In scenarios requiring robustness in complex noisy environments, when multiple types of noise such as motion artifacts and baseline drift exist, the detection accuracy of neural networks is significantly superior to that of traditional algorithms [115], and they can achieve more reliable heart rate monitoring while maintaining low power consumption.

In multi-feature joint detection tasks, when it is necessary to simultaneously detect morphological abnormalities of R-waves, P-waves, and T-waves, neural networks can realize multi-task processing with a single model through end-to-end training, whereas traditional algorithms need to combine multiple modules [108]. In personalized medical scenarios, when model fine-tuning for specific patients is required, neural networks can achieve adaptation by updating only the parameters of the final few layers through transfer learning [116], which is difficult for traditional algorithms to accomplish.

However, the parameter size and processing time of some neural networks vary depending on the structure of different neural networks. Some have more parameters, higher power consumption, and longer heart rate signal processing time. Therefore, for monitoring in long-term complex scenarios using flexible wearable heart rate systems, strongly quantized neural networks can be selected, such as lightweight Temporal Convolutional Network (TCN).

For example, Thorir et al. [117] proposed a lightweight Temporal Convolutional Network (TCN) specifically designed for ECG heart rate abnormality detection in flexible wearable systems through lightweight design. In terms of model architecture, when processing ECG signals, this TCN first expands the signal channels via a 1×1 convolutional layer. The residual blocks consist of two dilated convolutional layers, with batch normalization layers, non-linear activation layers, and dropout layers interspersed between them. By stacking these residual blocks, the receptive field of the model can be effectively expanded. The TCN proposed in this study features multiple low-power design characteristics: it has only 14,883 parameters, which is merely 1/27 of the parameter count of the comparable LSTM-FCN model. Its number of multiply-accumulate operations (MACs) is 1,030,260, accounting for only 1/37 of that of the LSTM-FCN, and the power consumption for one run is only 1/12 of that of the LSTM-FCN. This significantly reduces computational complexity and power consumption. In the residual blocks, weight normalization is replaced by batch normalization, and the batch normalization operation is performed at the 1 × 1 convolutional layer in the residual branch. Meanwhile, standard dropout is used instead of spatial dropout. These optimization measures avoid complex computations, and through calculations, it is known that the total system power consumption is approximately 1.8 mW. Compared with the existing Convolutional Neural Network (CNN) and Gated Recurrent Unit (GRU) solutions, the energy consumption is reduced by 19.6 times.

Although this study proposes a lightweight Temporal Convolutional Network (TCN), its long-term operational efficiency on energy-constrained devices still requires further evaluation. To this end, this paper collates and compares the computational overhead of this TCN with that of the Pan-Tompkins algorithm, Threshold Detection Algorithm, LSTM-FCN algorithm, and Lightweight Support Vector Machine (SVM) Classifier. The reason for selecting these algorithms for comparison is as follows: the Pan-Tompkins algorithm and Threshold Detection Algorithm are classic methods in the field of heart rate monitoring, characterized by simple computation and low power consumption, serving as important references for lightweight design; the Lightweight SVM Classifier is representative among traditional machine learning algorithms; and the LSTM-FCN algorithm represents the mainstream architecture in deep learning.

The comparison results are shown in Table 6. Data indicate that R-wave detection based on the Pan-Tompkins algorithm requires only 0.8 milliseconds of computation time per heartbeat, with a power consumption of 0.25 milliwatts [113]. In contrast, the inference process of the TCN model requires 12.5 milliseconds and 1.8 milliwatts [117], which are 15.6 times and 7.2 times those of the Pan-Tompkins algorithm, respectively. The Threshold Detection Algorithm has a signal processing time of 0.5 milliseconds, with a power consumption of 0.18 milliwatts [114]; while the Lightweight SVM Classifier has a signal processing time of 4.3 milliseconds and a power consumption of 0.75 milliwatts [118]. It is evident that the Pan-Tompkins algorithm, Threshold Detection Algorithm, and Lightweight SVM Classifier all outperform the lightweight TCN model in terms of signal processing time and power consumption. However, this is because the Pan-Tompkins algorithm, Threshold Detection Algorithm, and Lightweight SVM Classifier have relatively simple computational logic, without the need for a large number of parameters and complex calculations. Meanwhile, the Lightweight SVM Classifier is only applicable to scenarios with limited heart rate data, minimal noise interference, and single-task requirements [118], whereas the TCN is not only suitable for the aforementioned simple scenarios but also performs excellently in heart rate monitoring in complex environments and heart rate monitoring scenarios with multi-task objectives.

Finally, compared with the LSTM-FCN algorithm, which has a signal processing time of 58.4 milliseconds and a power consumption of 21.6 milliwatts [117], the lightweight TCN still has obvious advantages.

In summary, whether it is the low power consumption achieved by traditional heart rate calculation algorithms through coefficient optimization, precise setting of statistical ranges, and approximate computation, or the low power consumption accomplished by neural networks via structural simplification and model quantization, these methods collectively provide a wealth of effective strategies for the low-power design of algorithms used in flexible wearable heart rate signal computation. Meanwhile, it should be noted that although neural network algorithms such as TCN are sometimes inferior to traditional hard-coded algorithms like the Pan-Tompkins algorithm and Threshold Detection Algorithm and the Lightweight SVM Classifier in terms of power consumption and response time, they have greater advantages in heart rate monitoring in complex scenarios and multi-task target scenarios, with stronger robustness.

### 5.3. Hardware–Algorithm Co-Design for Flexible Wearable Heart Rate Signal Processing Module

Hardware–algorithm co-design is also a key solution for realizing heart rate estimation, arrhythmia classification, and low-power module design. This subsection will focus on how to achieve the functionality and low-power design of the heart rate calculation module through hardware–algorithm co-design.

Regarding heart rate estimation, Qiu et al. [119] through hardware–algorithm co-design, jointly developed a PPG-based power-consuming heart rate estimation processing module for flexible wearable systems. The system consists of a PPG Driver, preprocessing, downsampling, Data Buffer, FFT Module, and HR Estimation. The system adopts a customized processor with parallel computing capabilities and combines it with the R22SDF-FFT algorithm to achieve computational acceleration and reduce resource requirements. Meanwhile, by integrating the embedded processor with historical heart rate data records, the system minimizes and corrects heart rate estimation errors, thereby reducing the frequency of error generation and further lowering the system’s computational power consumption. Finally, validation on the SPC dataset [47] shows that the system exhibits a low mean absolute error in both 12-channel and 22-channel PPG recording sets [120], features short processing latency, and consumes only 34.7 µW of power.

Regarding arrhythmia classification, Janveja et al. [109] proposed a low-power arrhythmia classification module based on deep neural network (DNN) in flexible wearable systems through hardware–algorithm co-design. The ECG processing flow and the DNN design are illustrated in Figure 5c. At the hardware–algorithm co-design level, the system employs a streaming processing design, integrating filtering, R-peak extraction, and deep neural network (DNN) classification functions in hardware to eliminate power loss caused by data transmission between algorithms and hardware. Meanwhile, through dynamic power management, the finite state machine (FSM) controls the deep neural network (DNN) to enter standby mode when no effective heartbeat signal is detected, achieving a 40% reduction in power consumption. Furthermore, the system adopts a 180 nm bulk CMOS process to minimize leakage power, while a sequential shift multiplier is designed to replace the traditional multiplier. Multiply-accumulate operations are realized through shifting and a single 32-bit addition, reducing the number of registers. Concurrently, the input layer is simplified from 417 dimensions to 210 dimensions, avoiding redundant calculations caused by zero-filling and resulting in an average system power consumption of 8.75 µW. In terms of algorithm optimization, the system simplifies the R-peak extraction algorithm by only using difference comparison and zero-crossing detection, eliminating the need to store intermediate samples. Additionally, a lightweight network with a structure of 210 × 35 × 25 × 5 is constructed, where weights are quantized with 16-bit fixed-point numbers to avoid power consumption from floating-point operations. These hardware–algorithm co-design strategies collectively ensure low-power operation while maintaining efficient processing performance.

In summary, hardware–algorithm co-design has become a key approach for the low-power design of heart rate computation modules, integrating customized hardware (such as parallel processors and low-power CMOS) with optimized algorithms (such as lightweight DNN and efficient FFT). Case studies of Qiu’s PPG system and Janveja’s DNN classifier demonstrate that this approach achieves three core objectives: computational acceleration, high-precision estimation, and ultra-low-power consumption. Such co-design proves that low-power consumption and performance can be balanced, making it an ideal choice for next-generation flexible wearable systems.

The Table 7 lists the innovativeness, practicality, power consumption optimization methods, power consumption indicators of each technology, as well as the comparison of power consumption with previous studies, with specific contents shown in Table 7. Since the algorithm aspect of the heart rate signal computation module involves numerous algorithms, this table selects one traditional algorithm and one neural network algorithm as examples for list.

## 6. Design of the Flexible Wearable Heart Rate Signals Transmission and Output Module

After the heart rate signals are computed, they need to be stably and efficiently transmitted and output to terminal devices such as mobile phones and medical monitoring platforms through the heart rate signal transmission and output module for real-time viewing and further analysis. This process not only involves hardware-level selection and design—for example, wired transmission methods can adopt USB interface transmission, serial port transmission, etc., while wireless transmission can use modules such as Bluetooth Low Energy (BLE), Wi-Fi, and Zigbee and so on to ensure the stability and low-power characteristics of data transmission—but also relies on algorithm-level support, including data simplification through reliable compression algorithms, data integrity protection via verification algorithms, and power consumption reduction by optimizing data transmission protocols. Meanwhile, hardware–algorithm co-design is equally critical in this link; through rational allocation of hardware resources and algorithm functions, the low-power operation of the entire system can be achieved.

Next, this section will elaborate on the key points and implementation methods of low-power design for heart rate signal transmission and output from multiple dimensions, including hardware, algorithms, and hardware–algorithm co-design.

### 6.1. Low-Power Hardware Design for Flexible Wearable Heart Rate Signal Transmission and Output Module

In terms of hardware transmission for flexible wearable systems, both wired and wireless methods are commonly employed. Wired transmission methods include USB interface transmission, serial port transmission (UART) [121], or Ethernet port transmission via Ethernet. Wired transmission has advantages in stability, anti-interference performance, and data transmission rate, but it is limited by the wired connection mode, resulting in lower flexibility. Wireless transmission methods adopt modules such as BLE, Wi-Fi, and Zigbee and so on. The advantages of wireless transmission lie in high flexibility, convenient deployment, and adaptability to miniaturized designs; however, its drawbacks include transmission stability being affected by the environment and transmission rate sometimes being slower than that of wired transmission.

Given that wireless transmission can enhance the flexibility of flexible wearable heart rate monitoring systems, this section primarily takes wireless transmission as an example to introduce the low-power design of the transmission and output module in flexible wearable systems.

**Figure 6:**
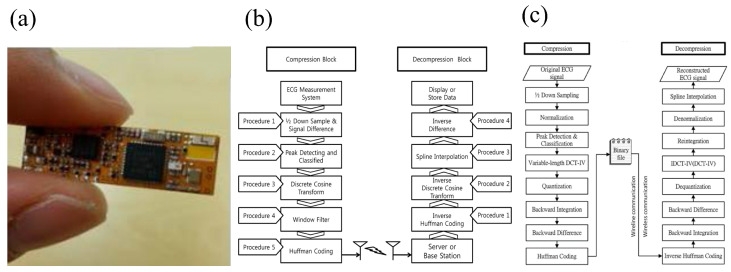
(**a**) Low-power Bluetooth module based on CC2541 chip [122]; this image is licensed under CC BY 4.0, copyright 2019, IEICE TRANSACTIONS on Information and Systems. (**b**) Program diagram of the DCT compression algorithm. Reproduced with permission [123], copyright 2011, IEEE. (**c**) Program diagram of the DCT-IV compression algorithm. Reproduced with permission [71], copyright 2016, IEEE.

For example, Jung et al. [122] developed a Bluetooth communication and output module for flexible wearable heart rate signal transmission with the low-power CC2541 chip as the core, whose structure is shown in Figure 6a. As the CC2541 chip itself consumes extremely low power, the module further reduces energy consumption by adopting a working–standby mode: it switches between working and standby states every 10 s to avoid prolonged high-power operation. Eventually, the overall power consumption of the Bluetooth communication module based on this chip can be controlled within 3 mW, fully meeting the low-power requirements for the long-term operation of flexible wearable systems.

In addition to the BLE module, for scenarios requiring longer transmission distances, Wi-Fi modules [124,125] can leverage their advantages of a high data rate and long-distance transmission for data relay, ensuring stable transmission of heart rate data from the collection end to remote servers or receiving devices.

For example, Shi et al. [126] developed a Wi-Fi-based flexible wearable heart rate cloud monitoring module through hardware selection and peripheral circuit design. The module reduces energy consumption at the core level by adopting low-power chips, and furthermore, the voltage regulators in peripheral circuits stabilize voltage and optimize current transmission, thus avoiding non-ideal operating states caused by voltage anomalies and directly reducing both dynamic and static power consumption. To further minimize module power consumption, the adopted MQTT protocol streamlines the protocol header and reduces communication overhead, making it suitable for networks with limited bandwidth and consequently lowering transmission energy consumption. It consumes more than 25% less power than traditional wired transmission and output.

Furthermore, the Zigbee module [127,128] demonstrates unique value in constructing complex heart rate monitoring networks by virtue of the dual advantages of its self-organizing network capability and low-power characteristics. The module adopts the IEEE 802.15.4 low-power protocol, which consumes only microampere-level current in sleep mode. Combined with a dynamic wake-up mechanism, it can activate the node communication function on demand. Its self-organizing network architecture supports multi-hop relay transmission, enabling the relay of heart rate signals among dozens of collection nodes without a fixed infrastructure. Meanwhile, the Carrier Sense Multiple Access with Collision Avoidance (CSMA/CA) mechanism avoids power consumption waste caused by collision retransmissions.

However, in scenarios with multiple users or crowded radio frequency (RF) environments, such as hospitals and gyms, wireless channels are vulnerable to co-channel interference, multipath fading, and system collisions, which may lead to interruptions or distortion in heart rate data transmission and conflict with the core goal of continuous monitoring. The specific manifestations include the following: packet loss: in dense deployments of BLE/Wi-Fi/Zigbee modules, channel competition can increase the loss rate of critical heart rate data [129]; increased transmission delay: interference-induced retransmission mechanisms prolong data transmission time, and if the delay exceeds the tolerance threshold of the monitoring system (real-time heart rate alerts require <500 ms) [130], it may affect the timeliness of clinical decision-making; and additional power consumption loss: frequent retransmissions or continuous channel scanning can increase the dynamic power consumption of wireless modules, and studies have shown that each additional retransmission can cause the power consumption of BLE modules to rise by 15–20% [131], conflicting with the goal of low-power design.

In response to the issues such as data packet loss, increased transmission delay, and additional power consumption loss caused by BLE, Wi-Fi, and Zigbee modules when radio frequency signals are congested, as mentioned in the aforementioned literature [122,124,125,126,127,128], this paper has collated and summarized a set of channel robustness optimization strategies for each module.

For BLE modules, which are prone to data loss due to 2.4 GHz frequency band conflicts in crowded RF scenarios, existing studies balance robustness and low-power consumption through two strategies. Adaptive frequency hopping (AFH) technology, such as the AFH mechanism supported by the CC2541 chip, can dynamically avoid interference bands such as channels interfered by MRI equipment in hospitals; by pre-setting frequency hopping maps to reduce channel scanning time, it lowers collision probability by 30% compared to fixed frequency hopping schemes, with additional power consumption controlled within 0.2 mW as hopping decisions rely on local channel quality detection without cloud interaction [132]. Dynamic transmission power adjustment, which adaptively reduces transmission power based on Receiver Signal Strength Indication RSSI at the receiving end, reduces interference to other systems while ensuring communication distance, lowering the average power consumption of BLE modules in crowded environments from 3 mW to 2.5 mW [133].

For Wi-Fi modules, to tackle interference issues under high loads, a low-power design needs to combine channel selection and protocol optimization. It can employ a real-time channel quality evaluation mechanism in its heart rate cloud monitoring module, identifying idle channels through intermittent monitoring; the switching process consumes only 0.5 mW of instantaneous power, reducing energy consumption by 60% compared to continuous channel scanning [134].

For Zigbee modules, which improve robustness through self-organizing network characteristics in complex monitoring networks while maintaining low-power consumption, the enhanced CSMA/CA mechanism introduces a “dynamic backoff window adjustment” strategy on the basis of traditional collision avoidance. When channel busy is detected, the backoff window expands exponentially to reduce invalid competition; during idle periods, the window shrinks quickly to lower waiting energy consumption, reducing the collision rate from 12% to 5% in 20-node networks with an average power consumption increase of no more than 0.3 mW [135].

In summary, in the low-power hardware design for the heart rate signal transmission and output module, BLE modules, Wi-Fi modules, and Zigbee modules each leverage their unique strengths. The BLE module extends system battery life while ensuring transmission quality through its low-power consumption and strong compatibility. The Wi-Fi module reduces power consumption via low-power chip features, MQTT protocol adoption, and peripheral circuit optimization, all while meeting long-distance transmission requirements. The Zigbee module, meanwhile, enables efficient and reliable transmission of heart rate signals among multiple nodes in complex monitoring networks through its self-organizing network capability. For issues such as data packet loss and transmission delay in complex scenarios, adaptive frequency hopping (AFH) technology, real-time channel evaluation mechanisms, and enhanced CSMA/CA mechanisms can also be adopted to solve the problems.

### 6.2. Low-Power Algorithm Design for Flexible Wearable Heart Rate Signal Transmission and Output Module

In the field of flexible wearable heart rate monitoring, the low-power design of algorithms for heart rate signal transmission and output is also of utmost importance. By applying efficient compression algorithms, such as Huffman coding, heart rate data can be accurately compressed to remove redundancy. At the same time, through the optimization of communication protocols, transmission parameters are dynamically adjusted. While ensuring the stable transmission of data, power consumption is effectively reduced, achieving the goal of low-power consumption at the algorithm level.

Generally speaking, data compression methods are divided into two categories: lossy compression and lossless compression. Lossy methods are widely used in the compression of electrocardiogram signals due to their high compression ratio performance. Previous research in this area can be classified into three categories. Firstly, calculate the redundancy of the signal and then remove the redundancy to obtain a higher data compression ratio. The second is the transformation method, which converts the time-domain signal into the frequency domain or other domains and compresses its spectrum or energy components. The third is the parameter extraction compression method, including the peak extraction method and the linear prediction method.

As reported by Lee et al., there is a DCT-based real-time ECG data compression and transmission algorithm for flexible wearable systems [123]. The system design is shown in Figure 6b. This algorithm is designed to address the challenges of efficient ECG data transmission and power consumption. The framework consists of five compression procedures and four reconstruction modules. During compression, the ECG signal undergoes 1/2 downsampling followed by backward difference computation. Subsequent peak detection in the differential signal partitions the data into segments for storage. The stored data then undergoes Discrete Cosine Transform (DCT), window-based filtering, and Huffman encoding for output. Reconstruction reverses these operations through Huffman decoding, inverse filtering, Inverse DCT (IDCT), and peak-point-based signal restoration.

On the basis of the research by Lee et al., Luo et al. innovatively reported a DCT-IV compression algorithm with variable transform length [71], which is used to improve the transmission efficiency of heart rate signals and reduce the power consumption of the flexible wearable system. The compression and decompression processes are shown in Figure 6c. The ECG compression algorithm reduces data processing complexity and computational energy consumption through backward difference calculation and non-uniform quantization, while its intelligent encoding of sign bits eliminates redundant operations. Compared with the traditional DCT-IV method, the algorithm effectively reduces data volume by 38.7% while suppressing the dynamic range of physiological signals. Meanwhile, the high compression ratio of the algorithm improves wireless transmission efficiency, thereby reducing communication energy consumption. For wearable cardiac monitoring devices, it can reduce the dynamic range requirement by 34.7% and keep the system power consumption below 5.0 mW, ensuring both energy efficiency and clinical value.

In addition to compressing heart rate signals using the above-mentioned optimized compression algorithms to reduce data redundancy and thus lower power consumption, power consumption can also be reduced by optimizing communication protocols. For example, adaptive modulation techniques [136,137,138] can be adopted to dynamically adjust transmission parameters according to channel conditions and data transmission requirements.

For example, Mukherjee et al. [139] proposed a signal transmission and output module based on the Dynamic Feedback-based Adaptive Modulation (DF-AM) algorithm to achieve the low-power design of flexible wearable heart rate systems. The algorithm optimizes the communication process by leveraging the time-varying correlation of wireless channels: the transmitter predicts the duration of channel states based on current conditions and triggers feedback only during state transitions, reducing overall feedback energy consumption by minimizing high-power feedback operations. Additionally, the DF-AM algorithm dynamically selects modulation indices according to real-time channel states, ensuring link adaptability while fully utilizing channel conditions to achieve low-power operation by design. When encountering harsh channel conditions, such as deep fading, the transmitter enters a power-saving mode to avoid energy waste from transmissions in unfavorable environments. The total power consumption of the system is approximately 60 mW. Compared with traditional Amplitude Modulation (AM) schemes, this system achieves over 22% energy savings and more than 13% throughput improvement.

This subsection focuses on the low-power algorithm design of flexible wearable heart rate signal transmission and output, adopting a “data streamlining + transmission optimization” strategy. It eliminates signal redundancy through compression and reduces ineffective transmission via adaptive modulation, thereby breaking through traditional power consumption limitations through their synergistic effect. For instance, the improved DCT-IV-based data compression algorithm optimizes dynamic range suppression, non-uniform quantization, and Huffman lossless coding. While maintaining accuracy, it achieves a compression ratio exceeding 25:1, significantly reducing the data volume for transmission. The DF-AM algorithm innovatively leverages channel time-domain correlation. By dynamically adjusting modulation indices and employing an intelligent feedback mechanism, it achieves a 22% energy efficiency improvement in complex wireless scenarios.

### 6.3. Hardware–Algorithm Co-Design for Flexible Wearable Heart Rate Signal Transmission and Output Module

In the heart rate signal transmission and output module, hardware–algorithm co-design serves as a crucial element for achieving both module functionality and low-power consumption. This integrated approach combines optimal low-power hardware platforms with compatible communication protocols to enable efficient data transmission. By incorporating CRC verification algorithms [140], it ensures complete and error-free data transfer, thereby realizing more efficient and stable heart rate data transmission. Furthermore, hardware–algorithm co-design demonstrates outstanding performance in optimizing module energy consumption.

For example, Wu et al. [62] constructed an IoT-based flexible wearable health monitoring transmission and output module through hardware–algorithm co-design.Specifically, the BLE module on the central board of the system is configured based on data transmission algorithms and MQTT protocol algorithms [141,142]. Equipped with a built-in ARM Cortex M0 processor, this module can perform preliminary processing on collected physiological data, reducing data volume before transmitting via BLE. This hardware–algorithm co-design approach not only enhances the efficiency of data transmission algorithms but also optimizes power consumption. In terms of power management, the algorithm is tailored to optimize the hardware of the power board. It implements a charging mode of constant current followed by constant voltage through a linear charge controller, while a low-power voltage divider circuit is used to monitor battery voltage. These measures effectively reduce the hardware power consumption of the power board and extend battery life. Furthermore, the system employs a state monitoring and decision algorithm that adjusts parameter configurations according to the current hardware status. This enables hardware components, such as sensors, to switch between working and standby modes, thereby achieving low-power operation of the entire system. The power consumption is 44.57 mW when all sensors are operating; 18.03 mW when only the PPG sensor is active.

For another example, Abidin et al. [121] developed a flexible wearable ECG signal transmission and output module through hardware–algorithm co-design, with the hardware–algorithm co-design primarily manifested in its transmit (Tx) and receive (Rx) working modes. Specifically, during transmission, the Gaussian Frequency Shift Keying (GFSK) modulation algorithm [143] is adopted to modulate and transmit data from the MCU, while during reception, coherent or non-coherent demodulation algorithms are employed to demodulate the radio frequency signals emitted by the low-power nRF24L01 transceiver. This two-fold approach not only significantly reduces the complexity of system data processing but also enhances energy efficiency through GFSK modulation, thereby lowering the overall system power consumption. Simultaneously, the system ensures transmission accuracy via a Cyclic Redundancy Check (CRC) mechanism, which helps minimize additional power consumption arising from error-induced retransmissions. Furthermore, the integrated design of the system reduces the number of external components, while the Arduino Nano microcontroller coordinates data processing and transmission. Through parameter tuning and mode switching, this microcontroller further optimizes power consumption, resulting in an average system power consumption of approximately 72.6 mW, ensuring that the system achieves high energy efficiency and maintains stable performance under low-power conditions.

In summary, both Wu et al.’s IoT module and Abidin et al.’s ECG signal transmission module have consistently implemented low-power concepts throughout their hardware–algorithm co-design for heart rate signal transmission and output modules. From selecting low-power components and designing optimized circuit architectures on the hardware side, to configuring multiple operational modes and adopting low-power transmission technologies in the algorithm, these design strategies have effectively reduced system energy consumption and extended device operational duration.

The Table 8 lists the innovativeness, practicality, power consumption optimization methods, power consumption indicators of each technology, as well as the comparison of power consumption with previous studies, with specific contents shown in Table 8. Meanwhile, in terms of the hardware design of the heart rate signal transmission and output module, since BLE modules and Wifi modules are more commonly used, references [122,126] have been selected for list; in the algorithm aspect, given the similarity between the DCT compression algorithm and the DCT-IV compression algorithm, only reference [71] has been selected for list.

It should be noted that references [62,71,121,122,123,126,127,128,139] in this section are all derived from flexible wearable systems.

In conclusion, in the field of heart rate signal transmission and output for flexible wearable systems, low-power design has formed a complete technical system through hardware selection, algorithm optimization, and hardware–algorithm co-design. At the hardware level, compared with wired transmission, wireless transmission modules such as BLE, Wi-Fi, and Zigbee modules have respectively constructed a multi-layered data transmission architecture with their low-power compatibility, long-distance transmission optimization, and self-organizing network capabilities. In terms of algorithms, from Lee et al.’s DCT compression to Luo et al.’s variable-length DCT-IV optimization, system power consumption is reduced through data redundancy elimination and dynamic range suppression. The DF-AM algorithm, relying on the time-varying characteristics of the channel to dynamically adjust modulation parameters, can reduce 22% of invalid transmission energy consumption in deep fading scenarios. Hardware–algorithm co-design breaks through the limitations of single technologies: Wu et al.’s IoT platform reduces 30% of data volume in the preprocessing stage and lowers transmission load through the collaboration of microprocessors and MQTT protocols; Abidin et al.’s ECG system integrates GFSK modulation with nRF24L01 transceivers and combines CRC check mechanisms to control retransmission power consumption within 5% of the total energy consumption. In the future, with the development of flexible electronics, the low-power design of heart rate signal transmission and output modules will evolve towards “adaptive-intelligent-miniaturized”.

## 7. Summary and Outlook

As an innovative integration of flexible electronics and biomedical engineering, flexible wearable heart rate monitoring systems have broken through the limitations of traditional monitoring devices by integrating core technologies, such as ECG and PPG. These systems provide efficient solutions for real-time, continuous, and non-invasive heart rate monitoring. This paper presents a comprehensive review of the system design, core technologies, module designs (including signal acquisition, preprocessing, computation, transmission, and output), as well as key challenges. Notably, it focuses on the universal application of low-power design concepts in hardware selection (e.g., flexible sensors, low-power chips), algorithm optimization (e.g., dynamic sampling rate adjustment, lightweight neural networks), and hardware–algorithm collaboration (e.g., compressive sampling, sleep modes).

In the design of the heart rate acquisition module for flexible wearable heart rate monitoring systems, piezoelectric materials, such as PVDF and piezoelectric ceramics, can be utilized. These materials can be employed to fabricate self-powered sensors. These sensors do not require external energy sources, thus achieving a low-power design. At the same time, the sampling frequency of the heart rate acquisition module is adaptively adjusted to achieve the optimal sampling frequency, thereby reducing the energy consumption of the module. For the design of the heart rate preprocessing module, multiple filter circuits can be integrated on a flexible FPC for signal preprocessing. Meanwhile, algorithms such as LMS filtering, wavelet filtering, and neural network algorithms are employed for heart rate signal preprocessing. By reducing noise in the signal through preprocessing, the computational load of the module is minimized, thereby lowering power consumption. In the design of the heart rate computation module, customizing the processor circuit makes the hardware more adapted to the calculation process, improving heart rate calculation efficiency. Combined with lightweight neural network algorithms, it enables more efficient heart rate calculation and reduces module energy consumption. For the design of the heart rate signal transmission module, using low-power Bluetooth modules, Wi-Fi modules, etc., can effectively reduce module power consumption at the hardware level. Meanwhile, matching with efficient compression algorithms reduces power consumption during signal transmission.

Looking to the future, the evolutionary path of flexible wearable technologies centered on low-power design is showing distinct features of multidisciplinary integration. At the hardware innovation level, flexible transistor arrays based on organic semiconductors [144] are expected to reduce power density to below 10 nW/mm^2^, with this material capable of stable operation over 100,000 cycles while maintaining a bending radius of 10 cm. Meanwhile, three-dimensional integration technology using low-temperature co-fired ceramic (LTCC) processes [145] can reduce the volume of signal conditioning circuits by 60%. When combined with energy harvesting modules (such as triboelectric nanogenerators), it enables all-weather self-powered monitoring. In circuit design, the combination of pulse width modulation (PWM) technology [146] and adaptive bias circuits dynamically adjusts the sampling frequency according to the amplitude of heart rate fluctuations, reducing the sampling rate to 128 Hz in a resting state and automatically increasing it to 512 Hz during exercise, thus achieving intelligent power distribution.

Algorithm-level innovations demonstrate the unique advantages of artificial intelligence technologies. A dynamic sampling strategy based on deep learning constructs an adaptive Bayesian model by analyzing the time-domain and frequency-domain distributions of historical heart rate data [147]. When the heart rate variability (HRV) index is detected to be within a stable range, it automatically reduces the data acquisition frequency from 256Hz to 64Hz, while proactively increasing the sampling frequency and activating an early warning mechanism at the onset of abnormal HRV fluctuations. Clinical trials have verified that this intelligent regulation strategy can reduce system power consumption by over 45% without affecting the extraction of key physiological features. More notably, the innovative model of hardware–algorithm co-design—customizing specialized accelerators with systolic arrays [148] for the computational characteristics of Wavelet Transform denoising algorithms—can shorten the algorithm execution time from 120 ms on traditional CPUs to 15 ms while reducing power consumption by 70%. This “tailor-made” design philosophy is becoming the core technical path to break through power consumption bottlenecks.

From the perspective of the ultimate form of technological evolution, future flexible wearable systems are expected to achieve breakthrough progress in the field of fully flexible devices: all electronic components, including resistors, capacitors, inductors, flexible thin-film transistors (TFTs) and so on, are projected to be manufactured with a micrometer-scale thickness. This will completely eliminate rigid components (such as the hard substrates of traditional silicon-based chips and metal pins), ultimately forming a fully bendable, stretchable, and even foldable “fully flexible system” [149,150].

However, the development of fully flexible wearable systems is not without challenges and still faces numerous obstacles.Here, explanations will be made with components such as resistors, capacitors, inductors, TFTs and so on.

At the material level, existing flexible materials have obvious drawbacks. The temperature resistance of flexible substrates (such as PDMS and PI) needs to be improved, which causes the resistance stability of flexible resistors and the dielectric properties of capacitors to be easily affected in high and low temperature environments. According to the properties of basic circuit components in the literature [151], it is assumed that the magnetic permeability of inductors and the switching characteristics of TFTs will also change. In addition, the compatibility between these flexible substrates and electronic materials is poor [152]. When combined with the conductive layers of flexible resistors and the dielectric layers of flexible capacitors, interface issues are likely to occur, leading to a decline in device performance. It is reasonably inferred that problems may also arise when flexible substrates are combined with flexible inductors or TFTs.

At the manufacturing process level, high-difficulty manufacturing technologies have become a bottleneck hindering the development of fully flexible wearable systems. Technologies such as the alignment of gate-source-drain electrodes for TFTs with micrometer-level thickness are still immature and cannot meet the requirements of high-performance devices [153]; meanwhile, the lead welding of flexible resistors and the magnetic core packaging of inductors have extremely high precision requirements, and any deviation may affect the normal operation of the devices. According to the literature [154], it can be reasonably inferred that the stacking alignment of capacitors and the multi-layer wiring of TFTs also have equally high precision requirements.

At the level of performance reliability, the stability of fully flexible structures faces severe challenges. Currently, the degradation mechanisms such as resistance drift of resistors, capacitance attenuation of capacitors, and threshold voltage shift of TFTs under repeated bending and stretching conditions in fully flexible structures are still unclear. According to the literature [155], it is reasonably inferred that other components, such as the Q-value reduction of inductors, may also occur; in addition, the packaging technology of fully flexible systems also needs innovation. Packaging materials must not only protect flexible resistors, capacitors, inductors, and TFTs from water, sweat, and corrosive environments but also maintain good flexibility to adapt to long-term wearing scenarios [156].

With the popularization of 5G IoT technology and the improvement of edge computing architectures, flexible wearable heart rate monitoring systems are evolving toward “perception-analysis-decision-making” integrated intelligent terminals. In the future, by combining with blockchain technology to achieve trusted medical data storage and integrating with brain–computer interface technology to build a mind–body collaborative monitoring network, these systems will not only serve as physiological signal acquisition tools but also become intelligent nodes connecting personal health management, family medical monitoring, and community health services.

## Figures and Tables

**Table 1:** Comparison between this paper and the existing literature on flexible wearable heart rate monitoring systems.

Comparison Dimensions	Reference [12]	Reference [30]	This Paper
Core topic	Analysis and summary of flexible wearable systems for cardiac physiological monitoring.	The Latest Progress and Applications of Flexible Wearable systems in Intelligent Health Monitoring.	Low-power Design of Flexible Wearable Heart Rate Monitoring System.
Low-power design	Merely mentioning the low-power requirements without elaborating on specific optimization strategies.	Merely mentioning low-power-related directions such as self-power supply and material optimization in the ‘Future Outlook’ section.	Low-Power Design of Acquisition, Preprocessing, Computation, and Transmission Modules from Three Dimensions: Hardware, Algorithms, and Hardware—Algorithm Collaboration.

**Table 2:** Key references comparison of innovations, practicality, power consumption optimization methods, power consumption and power consumption comparison with other similar work of regarding heart rate signal acquisition module in flexible Wearable Heart Rate Monitoring System.

Reference	Innovation	Practicality	Power Optimization Method	Power Consumption	Power Comparison
[42]	An ultrathin sensor based on electronic tattoo technology.	Achieve non-invasive and continuous physiological information monitoring.	Hardware	3 mW	Compared with traditional ECG sensors, the power consumption is reduced by more than 40%.
[81]	The first standalone skin-like patch integrating a stretchable OLED array with a PPG sensor.	Adhere to the skin and enable real-time heart rate display.	Hardware	16.5 mW	N/A
[68]	An optimal sampling rate optimization algorithm is proposed.	Reduce storage costs and extend device battery life.	Algorithm	N/A	Compared with traditional algorithms, the adaptive optimal sampling rate algorithm reduces system power consumption.
[63]	The first application of compressed sensing technology to a PPG-specific integrated circuit.	Achieve ultra-low power consumption, suitable for clinical research in health monitoring.	Hardware–Algorithm co-design	1.66 mW	Reduction of about 57% Compared with previous reports, the power consumption has been reduced by approximately 57%.
[80]	The system achieves low-power operation at a high sampling rate of 400 Hz.	Support 137 h of continuous monitoring and meets daily monitoring needs.	Hardware–Algorithm co-design	13.468 mW	Representing a 16.5% reduction in power consumption compared to systems without compression algorithms.

**Table 3:** Comparison of filtering algorithm performance under different motion states.

Filtering Algorithm	Resting State	Running State	Power Consumption (mW)
LMS Adaptive Filtering [94,95]	MSE = 0.021, SNR = 28.5 dB	MSE = 0.089, SNR = 20.1 dB	2.3
NLMS Adaptive Filtering [94,95]	MSE = 0.019, SNR = 29.2 dB	MSE = 0.078, SNR = 21.0 dB	2.5
Wavelet Filtering [96,97]	MSE = 0.017, SNR = 30.1 dB	MSE = 0.065, SNR = 22.3 dB	3.1
Kalman Filtering [96,97]	MSE = 0.023, SNR = 27.9 dB	MSE = 0.095, SNR = 19.5 dB	3.5

**Table 4:** Comparison of performance attenuation rates of various algorithms under different environmental conditions.

Environmental Variables	Performance Indicators	LMS Attenuation Rate (%) [98,99]	NLMS Attenuation Rate (%) [98,99]	WT Attenuation Rate (%) [96,100]	KF Attenuation Rate (%) [96,100]
Humidity Increased to 40% RH	THD	+85.7	+60.7	+25.0	+32.1
Temperature Increased to 35 °C	SNR	−18.2	−12.5	−8.7	−7.3
Increased Exercise Intensity	BD	+360	+240	+120	+150

**Table 5:** Key references comparison of innovations, practicality, power consumption optimization methods, power consumption and power consumption comparison with other similar work of regarding heart rate signal preprocessing module in flexible Wearable Heart Rate Monitoring System.

Reference	Innovation	Practicality	Power Optimization Method	Power Consumption	Power Comparison
[62]	RC network-optimized low-pass filter.	Suitable for signal preprocessing.	Hardware	10 μW	Compared with traditional filters, the power consumption is reduced by 40%.
[44]	Band-pass filter based on tunable pseudo-resistor.	Suitable for heart rate signal preprocessing.	Hardware	460 μW	Compared with traditional filters, the power consumption is reduced by 30%.
[69]	Optimizing the step size and filter order to enhance denoising performance.	Enhance the quality of ECG signals and provides support for heart rate monitoring.	Algorithm	N/A	Compared with the LMS algorithm, the NLMS algorithm has a faster convergence speed.
[99]	A neural network-based heart rate tracking method for motion scenarios.	Reduce computational complexity and is suitable for flexible wearable devices.	Algorithm	N/A	Compared with traditional filtering algorithms, it can more accurately suppress motion interference.
[106]	A method of using feature normalization preprocessing is proposed to improve recognition accuracy.	Suitable for mental health monitoring and human–computer interaction scenarios.	Hardware–Algorithm co-design	14.4 mW	Compared with the scenario where no normalization method is used, the preprocessing power consumption can be reduced by 25%.

**Table 6:** Comparison of computational overhead of different heart rate calculation algorithms.

Algorithm Type	Single Processing Time (ms)	Power Consumption (mW)	Number of Parameters
Pan–Tompkins Algorithm [113]	0.8	0.25	N/A
Threshold Detection Algorithm [114]	0.5	0.18	7
Lightweight SVM Classifier [118]	4.3	0.75	641
LSTM-FCN model [117]	58.4	21.6	404,741
Lightweight TCN (Temporal CNN) [117]	12.5	1.8	14,883

**Table 7:** Key references comparison of innovations, practicality, power consumption optimization methods, power consumption and power consumption comparison with other similar work of regarding heart rate signal computation module in flexible Wearable Heart Rate Monitoring System.

Reference	Innovation	Practicality	Power Optimization Method	Power Consumption	Power Comparison
[45]	A BioAIP processor for heart rate calculation is proposed.	Suitable for various wearable health monitoring scenarios.	Hardware	46.8 μW	Its energy consumption is at the lowest level among similar systems.
[67]	The ternary neural network processor for ECG signal computation is proposed for the first time.	Suitable for early warning of cardiac arrhythmias.	Hardware	746 nW	A power consumption reduction of more than 71.6% compared to traditional heart rate calculation is achieved.
[111]	A low-power statistical FFT signal analysis method.	Suitable for heart rate calculation.	Algorithm	2.4 mW	Compared with the traditional FFT signal analysis algorithm, the power consumption can be reduced by more than 40%
[117]	A lightweight TCN model for heart rate calculation is proposed.	Suitable for wearable arrhythmia detection scenarios.	Algorithm	1.8 mW	Compared with existing CNN + GRU solutions, the energy consumption is reduced by 19.6 times.
[119]	A customized PPG heart rate estimation hardware has been developed.	Suitable for high-precision long-term heart rate calculation and monitoring.	Hardware–Algorithm co-design	34.7 μW	Compared with microcontroller solutions, it reduces power consumption from the milliwatt level to the microwatt level.
[109]	A low-power ECG coprocessor based on DNN is proposed.	Suitable for real-time monitoring scenarios in wearable devices.	Hardware–Algorithm co-design	8.75 μW	Compared with similar solutions, the Figure of Merit is improved by 3.48 times.

**Table 8:** Key references comparison of innovations, practicality, power consumption optimization methods, power consumption and power consumption comparison with other similar work of regarding heart rate signal transmission and output module in flexible Wearable Heart Rate Monitoring System.

Reference	Innovation	Practicality	Power Optimization Method	Power Consumption	Power Comparison
[122]	A flexible wireless sensor patch adopting BLE communication technology.	Enable real-time wireless transmission and monitoring of heart rate.	Hardware	Below 3 mW	Compared with similar devices, its power consumption is reduced by approximately 40%.
[126]	A Wi-Fi-based flexible wearable heart rate cloud monitoring module.	Suitable for wireless transmission of heart rate signals.	Hardware	N/A	Compared with traditional wired transmission and output, it consumes more than 25% less power.
[71]	A DCT-IV-based ECG compression algorithm is proposed.	Suitable for continuous heart rate monitoring.	Algorithm	Below 5 mW	N/A
[139]	A dynamic feedback adaptive modulation (DF-AM) scheme is proposed.	Suitable for low-power communication scenarios in the IoT.	Algorithm	60 mW	Compared with traditional AM schemes, this scheme achieves more than 22% energy savings.
[62]	A heart rate monitoring system with wireless transmission functionality.	Suitable for remote health monitoring scenarios and supports long-term wearability.	Hardware–Algorithm co-design	44.57 mW	Compared with similar devices, the power consumption is reduced by approximately 14.3%.
[121]	A three-lead portable wireless transmission heart rate monitoring system is developed.	Suitable for remote heart rate monitoring.	Hardware–Algorithm co-design	72.6 mW	N/A
